# CGEF-1 regulates mTORC1 signaling during adult longevity and stress response in *C. elegans*

**DOI:** 10.18632/oncotarget.24039

**Published:** 2018-01-06

**Authors:** Yujie Li, Sandra Finkbeiner, Athina Ganner, Julia Gerber, Marinella Klein, Manuel Grafe, Jakob Kandzia, Antje Thien, Kathrin Thedieck, Gerhard Breves, Thomas Jank, Ralf Baumeister, Gerd Walz, Elke Neumann-Haefelin

**Affiliations:** ^1^ Department of Nephrology, Medical Center, University of Freiburg, Freiburg, Germany; ^2^ Bioinformatics and Molecular Genetics, Faculty of Biology, Albert-Ludwigs-University Freiburg, Freiburg, Germany; ^3^ Department of Pediatrics, Section Systems Medicine of Metabolism and Signaling, University of Groningen, University Medical Center Groningen, Groningen, The Netherlands; ^4^ Department for Neuroscience, School of Medicine and Health Sciences, Carl von Ossietzky University Oldenburg, Oldenburg, Germany; ^5^ Institute for Experimental and Clinical Pharmacology and Toxicology, Faculty of Medicine, Albert-Ludwigs-University of Freiburg, Freiburg, Germany; ^6^ Centre for Biological Signaling Studies (BIOSS), Albert-Ludwigs-University of Freiburg, Freiburg, Germany; ^7^ Centre for Biological Systems Analysis (ZBSA), Albert-Ludwigs-University Freiburg, Freiburg, Germany; ^8^ ZBMZ Centre for Biochemistry and Molecular Cell Research, Faculty of Medicine, Albert-Ludwigs-University Freiburg, Freiburg, Germany; ^9^ Department of Physiology, University of Veterinary Medicine Hannover, Hannover, Germany

**Keywords:** mTORC1 signaling, Rheb, aging, stress response, C. elegans

## Abstract

The mechanistic target of rapamycin (mTOR) kinase is central to metabolism and growth, and has a conserved role in aging. mTOR functions in two complexes, mTORC1 and mTORC2. In diverse eukaryotes, inhibition of mTORC1 signaling increases lifespan. mTORC1 transduces anabolic signals to stimulate protein synthesis and inhibits autophagy. In this study, we demonstrate that CGEF-1, the *C. elegans* homolog of the human guanine nucleotide exchange factor Dbl, is a novel binding partner of RHEB-1 and activator of mTORC1 signaling in *C. elegans*. *cgef-1* mutants display prolonged lifespan and enhanced stress resistance. The transcription factors DAF-16/FoxO and SKN-1/Nrf are required for increased longevity and stress tolerance, and induce protective gene expression in *cgef-1* mutants. Genetic evidence indicates that *cgef-1* functions in the same pathway with *rheb-1*, the mTOR kinase *let-363*, and *daf-15*/Raptor. When *cgef-1* is inactivated, phosphorylation of 4E-BP, a central mTORC1 substrate for protein translation is reduced in *C. elegans*. Moreover, autophagy is increased upon *cgef-1* and mTORC1 inhibition. In addition, we show that in human cells Dbl associates with Rheb and stimulates mTORC1 downstream targets for protein synthesis suggesting that the function of CGEF-1/Dbl in the mTORC1 signaling pathway is evolutionarily conserved. These findings have important implications for mTOR functions and signaling mechanisms in aging and age-related diseases.

## INTRODUCTION

The mechanistic target of rapamycin (mTOR) kinase is a highly conserved and fundamental regulator of growth, metabolism, and aging in all eukaryotes [1, 2]. mTOR is present in two structurally and functionally distinct complexes, mTORC1 and mTORC2, which are defined by their association with Raptor and Rictor, respectively. mTORC1 promotes growth-related cellular processes such as protein synthesis and ribosome biogenesis, and inhibits autophagy. Diverse extra- and intracellular stimuli activate mTORC1 signaling including growth factors (e.g. insulin), amino acids, and energy sufficiency. A key upstream activator of mTORC1 is the small GTPase Rheb (Ras homolog enriched in brain) which directly associates with mTORC1 and is able to stimulate its kinase activity [3, 4]. Activation of mTORC1 is inhibited by the mammalian tuberous sclerosis complex (comprised of Tsc1 and Tsc2) which acts as a GTPase-activating protein (GAP) on Rheb [5, 6]. Growth factors are important stimuli of the Akt kinase that directly phosphorylate and inactivate the Tsc1/Tsc2 complex, thereby de-repressing Rheb and promoting mTORC1 signaling. Recent studies indicate that Rheb-mediated activation of mTORC1 takes place at the lysosomal membrane [7–9].

mTORC1 balances anabolic and catabolic cellular processes. Under favorable conditions rich in nutrients and energy mTORC1 stimulates anabolic processes including the synthesis of proteins and lipids [10]. Upon activation mTORC1 phosphorylates a set of well-characterized targets, most notably the ribosomal S6 kinase (S6K) and the translational initiation factor 4E-binding protein (4E-BP1) to promote protein synthesis. While stimulating the synthesis of macromolecules, mTORC1 inhibits catabolic processes like autophagy, a cellular recycling mechanism [10].

mTORC1 signaling plays an important role in the regulation of cellular senescence and organismal aging [1]. Genetic suppression or pharmacological inhibition of mTOR by rapamycin extends lifespan in diverse model organisms ranging from yeast and *C. elegans* to mammals [11]. Notably, in *C. elegans* mTOR signaling plays a fundamental role in the regulation of growth and longevity [12–15]. Importantly, post-developmental inhibition of mTORC1 or rapamycin treatment cause lifespan extension in adult *C. elegans* and mice [16, 17] suggesting that inactivation of mTORC1 signaling might be a promising anti-aging strategy. Knockdown of mTORC1 also enhances tolerance against environmental stress consistent with a shift towards tissue maintenance. Previous work has identified cellular processes through which mTORC1 affects lifespan. Reduction of protein synthesis has emerged an important mTORC1-regulated mechanism for longevity. A low level of translation under unfavorable stress conditions might be beneficial because of notable energy savings, but further evidence indicates that protective mechanisms are also mobilized [18]. Genetic studies in diverse model organisms have demonstrated that inhibition of genes involved in translation initiation or ribosomal biogenesis promote longevity [19–21]. In addition to reducing mRNA translation, *C. elegans* mTORC1 inhibition exerts a positive effect on lifespan in part through activation of the transcription factors DAF-16/FoxO and SKN-1/Nrf2 [16]. DAF-16 and SKN-1 regulate genes that protect against environmental, metabolic, and proteotoxic stress, and contribute to longevity [20]. Moreover, longevity effects conferred by mTORC1 inhibition require autophagy [22, 23].

Aberrant mTOR signaling has been implicated in a large number of age-related diseases including cancer, neurodegenerative syndromes, and cardiovascular diseases [1]. Although considerable insights into the regulation of mTORC1 have been made, many unresolved and poorly understood issues remain. In this study, we identify the guanine nucleotide exchange factor CGEF-1 as a novel binding partner of *C. elegans* RHEB-1. CGEF-1 facilitates mTORC1 signaling to control aging, stress response, protein synthesis, and autophagy in *C. elegans*. DAF-16/FoxO and SKN-1/Nrf exert protective gene expression and increase stress tolerance and longevity when *cgef-1* is genetically inhibited. Dbl, the homolog of CGEF-1 in mammals, associates with Rheb and promotes mTORC1 downstream signaling suggesting an evolutionary conserved function of Dbl/CGEF-1 in the mTOR pathway.

## RESULTS

### CGEF-1 interacts and functions in the same pathway with RHEB-1 to regulate longevity and stress response in *C. elegans*

**Figure 1:**
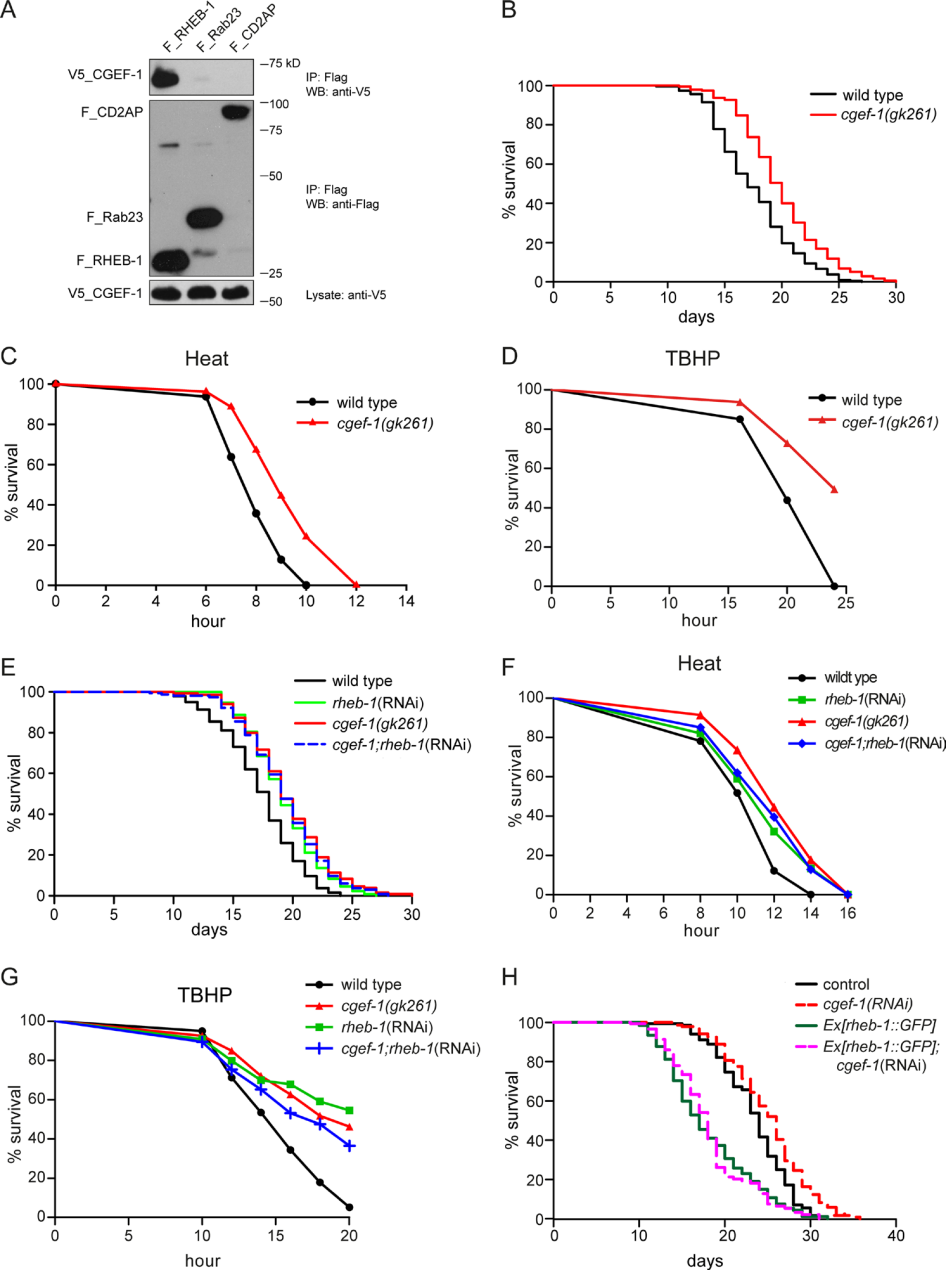
CGEF-1 is a novel RHEB-1 binding partner and functions in the same pathway with RHEB-1 to regulate longevity and stress response in C. *elegans* (**A**) *C. elegans* CGEF-1 interacts with RHEB-1 in HEK 293T cells. After immunoprecipitation (IP) with anti-Flag antibody, the immobilized CGEF-1 was detected by western blot (WB) analysis using anti-V5 antibody (upper panel). The control proteins Rab23 and CD2AP failed to bind CGEF-1. Middle part shows immunoprecipitation of Flag-tagged proteins, the lower panel shows the expression of V5-tagged CGEF-1 in cell lysates. kD, kiloDalton. (**B**) Lifespan of *cgef-1(gk261)* mutants is increased relative to wild type N2. Survival analysis represents the combined data from three experiments. See Table 1 for statistical analyses. (**C**) Heat stress resistance is increased in *cgef-1(gk261)* mutants compared to wild type N2. (**D**) *cgef-1* mutants show enhanced tolerance of oxidative stress from *tert*-butyl hydroperoxide (TBHP). (**E**) *cgef-1* functions in the same pathway with *rheb-1*. Longevity of *cgef-1(gk261)* mutants is not altered by *rheb-1*(RNAi) knockdown. Combined data from two experiments are shown. Statistics are available in Table 1. (**F**) Inhibition of *rheb-1* by RNAi does not affect heat tolerance of *cgef-1(gk261)* mutants. (**G**) The oxidative stress resistance conferred by mutation of *cgef-1* is not enhanced by *rheb-1*(RNAi) knockdown. Heat survival assays (C and F) were performed at 35°C. Data from a representative experiment are shown. Replicates and statistics are available in Supplementary Table 2. In the oxidative stress experiments (D and G) worms were exposed to 7.5 mM TBHP. A representative experiment is shown. See Supplementary Table 3 for replicates and statistics. (**H**) Overexpression of *rheb-1* suppresses *cgef-1*(RNAi) longevity. Combined data from two experiments are shown. Statistics are available in Table 1. The effectiveness of knockdown of *cgef-1* and *rheb-1* by RNAi was analyzed by feeding the RNAi plasmid to transgenic strains expressing a translational fusion of GFP to *cgef-1* or *rheb-1* respectively (Supplementary Figure 3).

Rheb is indispensable for the activation of mTORC1 signaling by amino acids and growth factors. To identify new regulators of RHEB-1 in *C. elegans*, we performed a yeast split-ubiquitin two-hybrid screen using full length RHEB-1 as bait. In this screen, CGEF-1 was isolated as a potential binding partner of *C. elegans* RHEB-1. *cgef-1* belongs to the family of Dbl proteins and encodes a *g*uanine nucleotide *e*xchange *f*actor (GEF). Interestingly, alterations of mammalian Dbl function lead to tumor formation and metastasis. We first aimed to confirm the interaction between RHEB-1 and CGEF-1 by co-immunoprecipitation from HEK 293T cells. Indeed, V5-tagged CGEF-1 co-precipitated with Flag-tagged *C. elegans* RHEB-1 but not with control proteins (Figure 1A) supporting that *C. elegans* RHEB-1 and CGEF-1 physically interact.

*cgef-1* exists in three isoforms with a common C-terminus and different N-termini (Supplementary Figure1A). To analyze the expression pattern of these different *cgef-1* isoforms we used GFP reporter fusions. *Pcgef-1*::GFP was broadly expressed in many tissues including the intestine, the vulva area, and some of the head and tail neurons (Supplementary Figure 1B). This expression pattern resembled the expression of *rheb-1*::GFP (Supplementary Figure 1C) and *let-363/CeTOR*::GFP (Supplementary Figure 1D) supporting the idea that RHEB-1 and CGEF-1 might interact to regulate mTORC1 in *C. elegans.* We also tested whether *cgef-1* is critical for the expression of *rheb-1*. No obvious alteration of RHEB-1::GFP was observed when *cgef-1* was knocked down by RNAi suggesting that *cgef-1* does not influence the expression of *rheb-1* (Supplementary Figure 1C).

We hypothesized that CGEF-1 may function in RHEB-1 signaling. If CGEF-1 acts with RHEB-1 in a common pathway one would expect to observe similar phenotypes when inhibiting either *cgef-1* or *rheb-1* function. As knockdown of *rheb-1* results in extension of lifespan and improved stress tolerance [16], we investigated the behavior of a *cgef-1* mutant in lifespan and stress assays. The *cgef-1* mutant *gk261* is a reported null allele that bears a deletion of 318 bp disrupting the GEF domain [24] (Supplementary Figure 1A). Mutation of *cgef-1* led to a moderate but significant increase of lifespan compared to wild type (+13 % lifespan extension) (Figure 1B and Table 1). This effect was similar to what we observed when *rheb-1* was knocked down by RNAi (+11 %; Table 1). *cgef-1* mutants also displayed enhanced resistance towards heat (Figure 1C) and the oxidizing agent *tert*-butyl hydroperoxide (TBHP) (Figure 1D). To exclude developmental effects of *cgef-1* inactivation on lifespan and stress phenotypes, we tested whether inhibition of *cgef-1* function by RNAi only during adulthood affects longevity. Indeed, adulthood *cgef-1*(RNAi) resulted in extended lifespan (Supplementary Figure 2A and Table 1) and enhanced heat and oxidative stress tolerance compared to control (Supplementary Figure 2B and 2C).

**Table 1:** Lifespan analyses

Strain	RNAi	mean lifespan ± SEM	median	75 %ile	*P* value vs N2	*P* value vs control	% change	*N*	No of exp	Figure
N2		17.6 ± 0.2	17	20				216/235	3	1B
cgef-1(gk261)		19.9 ± 0.2	20	22	< 0.0001		+13	185/227	3	1B
N2	control	18.5 ± 0.2	18	21				216/231	3	S2A
N2	cgef-1	21.0 ± 0.3	21	23	< 0.0001		+14	198/219	3	S2A
N2	control	17.3 ± 0.3	18	20				136/142	2	1E
cgef-1(gk261)	control	19.5 ± 0.3	19	22	< 0.0001		+13	133/144	2	1E
N2	rheb-1	19.2 ± 0.3	19	21	< 0.0001		+11	133/152	2	1E
cgef-1(gk261)	rheb-1	19.2 ± 0.3	19	22	< 0.0001	ns^a^; ns^b^	+11	135/157	2	1E
Ex[unc-119]	control	23.4 ± 0.3	24	26				130/138	2	1H
Ex[rheb-1::GFP, unc-119(+)]	control	18.1 ± 0.5	17	22	< 0.0001		-23	110/126	2	1H
Ex[unc-119]	cgef-1	25.2 ± 0.4	26	28	< 0.001		+7	128/142	2	1H
Ex[rheb-1::GFP, unc-119(+)]	cgef-1	18.3 ± 0.4	18	20	< 0.0001	ns^i^	-22	105/126	2	1H
N2	control	17.8 ± 0.2	18	20				216/224	3	2A, B
cgef-1(gk261)	control	20.2 ± 0.2	20	22	< 0.0001		+13	205/219	3	2A, B
N2	daf-15	21.1 ± 0.2	21	23	< 0.0001		+19	211/237	3	2B
cgef-1(gk261)	daf-15	21.7 ± 0.3	22	25	< 0.0001	ns^c^	+22	205/230	3	2B
N2	let-363	21.9 ± 0.3	22	25	< 0.0001		+23	210/238	3	2A
cgef-1(gk261)	let-363	21.7 ± 0.3	22	25	< 0.0001	ns^d^	+22	208/233	3	2A
N2	control	20.1 ± 0.2	20	23				195/219	3	3A, B, C
N2	cgef-1	23.5 ± 0.3	24	26	< 0.0001		+17	186/217	3	3A, B, C
skn-1(zu67)	control	15.5 ± 0.2	15	17	< 0.0001		−23	160/199	3	3B
skn-1(zu67)	cgef-1	17.9 ± 0.2	18	20	< 0.0001	< 0.0001^e^; < 0.0001^h^	−11	163/204	3	3B
daf-16(mgDf47)	control	18.0 ± 0.3	18	21	< 0.0001		−10	186/218	3	3A
daf-16(mgDf47)	cgef-1	17.8 ± 0.3	18	21	< 0.0001	ns^f^	−11	175/214	3	3A
daf-16;skn-1	control	14.3 ± 0.2	14	16	< 0.0001		−29	162/205	3	3C
daf-16,skn-1	cgef-1	14.4 ± 0.2	15	17	< 0.0001	ns^g^	−28	183/207	3	3C

These combined results were derived from individual experiments described in Supplementary Table 1. Lifespan extensions correspond to parallel N2 control experiments. N represents total number of animals dying of old age versus those in total experiment. SEM = standard error of the mean. ns = not significant.

*p*-values (log-rank test) refer to the following control experiments:

^a^ cgef-1(gk261);control(RNAi),

^b^ rheb-1(RNAi),

^c^ daf-15/Raptor(RNAi),

^d^ let-363/CeTOR(RNAi),

^e^ skn-1(zu67);control(RNAi),

^f^ daf-16(mgDf47);control(RNAi),

^g^ daf-16(mgDf47);skn-1(zu67);control(RNAi),

^h^ cgef-1(RNAi),

^i^ Ex[rheb-1::GFP;unc-119(+)];control(RNAi).

To test for a genetic interaction between *cgef-1* and *rheb-1*, we analyzed the effect of *rheb-1* knockdown in *cgef-1* mutants. We found that *rheb-1*(RNAi) did not further increase the longevity of *cgef-1(gk261)* mutants (Figure 1E and Table 1). Moreover, heat as well as oxidative stress resistance of *cgef-1* mutants was not further enhanced by *rheb-1*(RNAi) (Figure 1F and 1G). These findings indicate that *cgef-1* and *rheb-1* are epistatic. Additionally, we found that the lifespan extending effect associated with inhibition of *cgef-1* could be suppressed by overexpression of *rheb-1* (Figure 1H). Together, our data suggest that *cgef-1* functions in the same pathway with *rheb-1* to regulate lifespan and stress response in *C. elegans*.

### CGEF-1 promotes mTORC1 signaling

**Figure 2:**
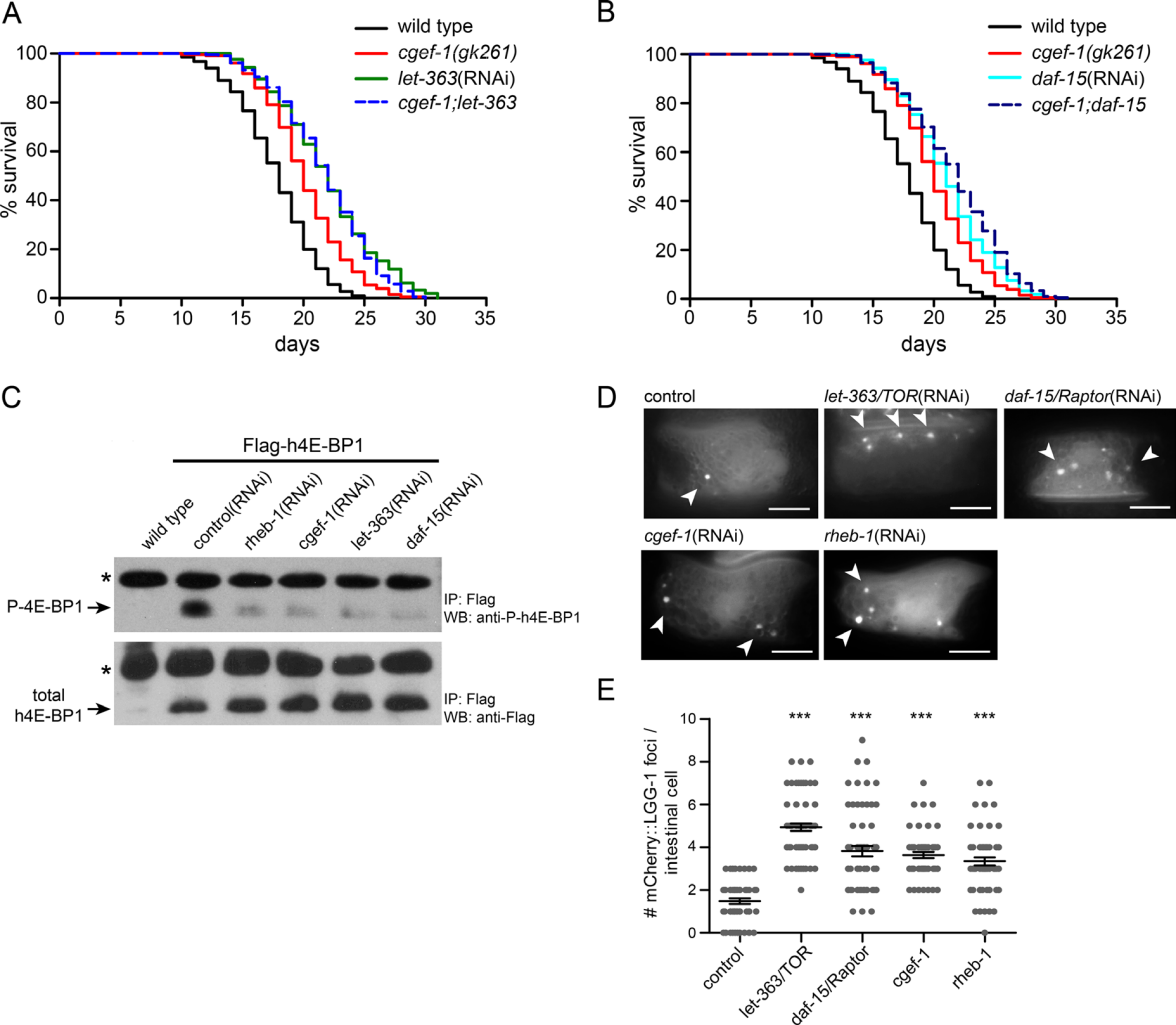
*cgef-1* functions in the mTORC1 signaling pathway (**A**) Longevity of *let-363/CeTOR*(RNAi) was not affected by the *cgef-1(gk261)* mutation. (**B**) Survival of *daf-15/*Raptor(RNAi) was not altered by *cgef-1(gk261)* mutation. For lifespan analysis (in A and B) *cgef-1(gk261)* and wild-type N2 worms were fed with *let-363*/*CeTOR*, *daf-15*/Raptor, or *control*(RNAi). Survival plots represent the combined data from three experiments. See Table 1 for statistical analyses. (**C**) CGEF-1 activates mTORC1 to phosphorylate the elongation factor 4E-BP1. Phosphorylation of transgenically expressed human 4E-BP1 was used to assess the activity of mTORC1 signaling. Worms carrying the *Pges-1::Flag-h4E-BP1* transgene were subjected to RNAi treatment as indicated. After immunoprecipitation (IP) with anti-Flag antibody the Flag-tagged h4E-BP1 was detected by western blot (WB) analysis (lower panel). Phosphorylation of h4E-BP1 was analyzed using anti-P-4E-BP antibody (upper panel). Star indicates antibody light chains. (**D**) Autophagy is increased after *cgef-1* and mTORC1-pathway gene knockdown. Representative pictures of L4 animals expressing mCherry::LGG-1 in the intestine. In control animals mCherry::LGG-1 is diffusely distributed in the cytosol. After inhibition of *cgef-1*, *rheb-1, let-363/CeTOR*, and *daf-15/*Raptor by RNAi an induction of mCherry::LGG-1-positive foci was observed (arrowheads). Scale bar represents 10 μm. (**E**) Quantification of mCherry::LGG-1 foci in intestinal cells in L4 larvae. Individual data points are plotted to illustrate variability. Bars indicate the mean and SEM for each genotype. *n* > 25 animals per each condition. ^***^p < 0.001 versus control, ANOVA.

Next, we assessed the genetic interaction between *cgef-1* and mTORC1 signaling. We used RNAi to knock down the mTOR kinase *let-363* and the mTORC1-specific gene *daf-15*/Raptor in *cgef-1* mutants and wild-type animals. Knockdown of each mTORC1 gene that we examined strongly increased lifespan in wild type (Figure 2A and 2B, Table 1). Mutation of *cgef-1* did not further enhance the longevity. Notably, the mean lifespan associated with *let-363*/*CeTOR*(RNAi) and *daf-15*/Raptor(RNAi) was similar in wild-type animals and *cgef-1* mutants (Figure 2A and 2B). Thus, we conclude that *cgef-1* acts in the same pathway with mTORC1 to regulate longevity. mTORC1 is also important for growth and development. While mutation of *cgef-1* had no effect on body size (Supplementary Figure 4A), inactivation of *cgef-1* led to a reduced brood size (Supplementary Figure 4B).

In mammals, mTORC1 signaling promotes growth by increasing protein synthesis through phosphorylation of 4E-BP and S6K and by inhibiting catabolic processes such as autophagy [10]. Monitoring the phosphorylation state of the mTORC1 substrate 4E-BP has been previously utilized to characterize mTORC1 activity in *C. elegans* [25]. Here, we took advantage of this readout and expressed human 4E-BP1 (h4E-BP1) tagged with Flag under control of the *ges-1* promoter in the intestine of *C. elegans*. We observed that *let-363/CeTOR*(RNAi) and *daf-15/*Raptor(RNAi) knockdown almost completely abolished the phosphorylation of h4E-BP1 (Figure 2C) indicating that human 4E-BP1 serves as substrate for *C. elegans* mTORC1. Interestingly, RNAi against *cgef-1* and *rheb-1* mimicked these effects and blocked h4E-BP1 phosphorylation to the same level as caused by *let-363*(RNAi) or *daf-15*(RNAi) (Figure 2C). These results indicate that CGEF-1 serves as an upstream activator for mTORC1 signaling.

We next wondered whether *cgef-1* also regulates autophagy, another mTORC1 downstream process. LGG-1, the worm ortholog of the yeast Atg8 and mammalian MAP1-LC3, localizes to autophagosomal membranes and has been widely deployed as an indicator of autophagy in *C. elegans* [22, 26]. Here, we used a reporter strain expressing mCherry-tagged LGG-1 in the intestine [27]. Upon induction of autophagy, mCherry::LGG-1 changes its diffuse cytoplasmic distribution pattern to punctate structures reflecting LGG-1 sequestration to autophagosomal membranes. In agreement with published data [16, 22], we observed a significant increase of mCherry::LGG-1 positive foci in intestinal cells in *let-363/CeTOR*(RNAi) and *daf-15/*Raptor(RNAi) treated animals compared to wild type (Figure 2D and 2E). An induction of mCherry::LGG-1 foci was also observed upon *rheb-1* and *cgef-1*(RNAi) knockdown indicating an increase in autophagy levels (Figure 2D and 2E). Taken together, we conclude that CGEF-1 associates with RHEB-1 and activates mTORC1 signaling to inhibit autophagy in *C. elegans*.

### Lifespan extension and enhanced stress resistance of *cgef-1* require the function of the transcription factors DAF-16/FoxO and SKN-1/Nrf2

**Figure 3:**
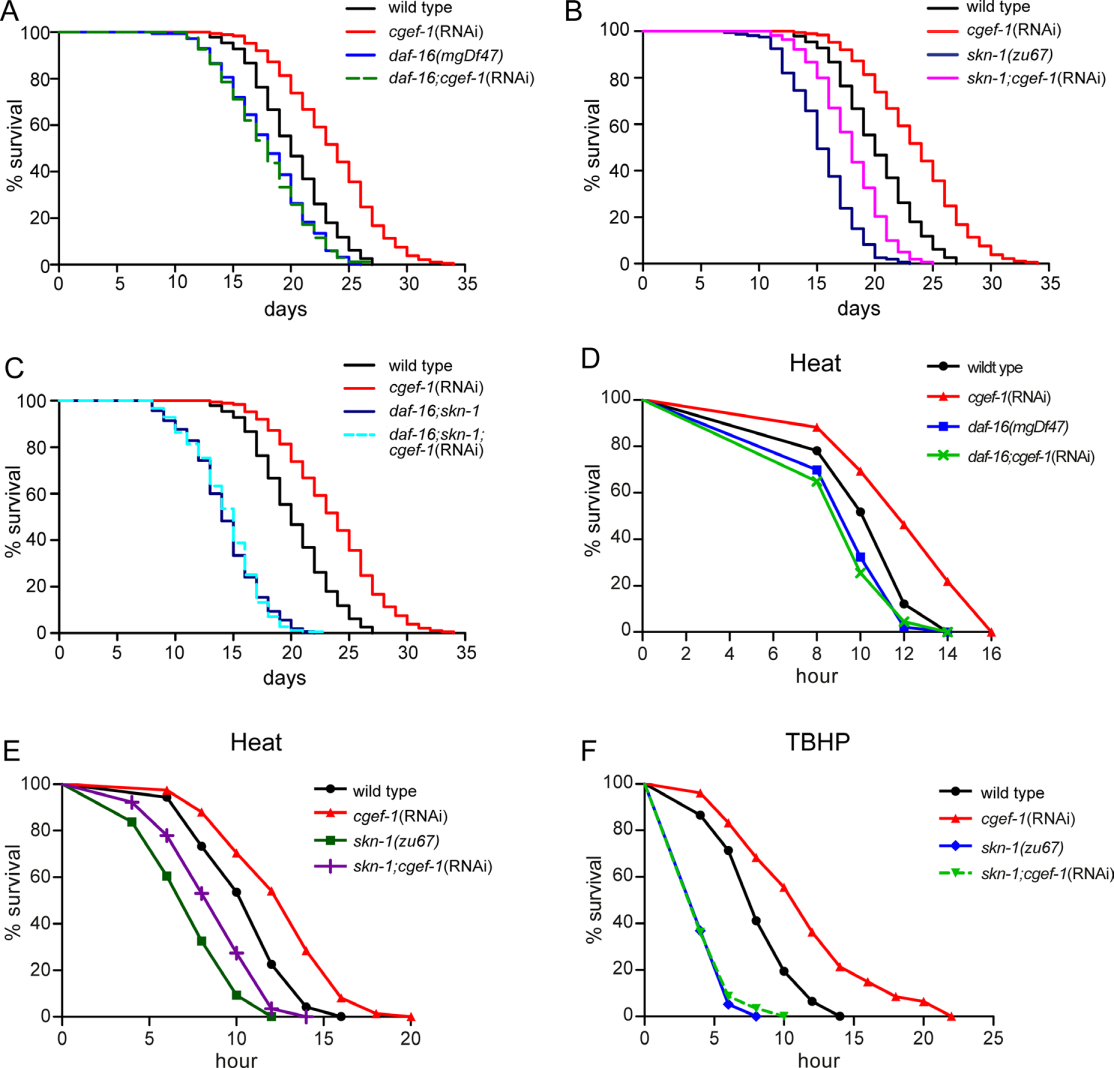
*cgef-1* regulates lifespan and stress response through the transcription factors DAF-16/FoxO and SKN-1/Nrf2 (**A**) Lifespan extension deriving from loss of *cgef-1* function requires *daf-16* function. (**B**) *cgef-1*(RNAi) extends lifespan in *skn-1* mutants. (**C**) Lifespan of *daf-16;skn-1* double mutants is unaffected by *cgef-1*(RNAi). In the lifespan experiments shown in (A–C), wild type N2, *daf-16(mgDf47), skn-1(zu67),* or *daf-16(mgDf47);skn-1(zu67)* mutants were fed with *cgef-1*(RNAi) or *control*(RNAi). Survival plots show composites from three experiments. See also Table 1 for statistics. (**D**) *cgef-1*(RNAi) increases resistance to heat (35°C) dependent upon *daf-16* function. (**E**) *skn-1* is not required to protect against heat stress. (D-E) Wild type N2, *daf-16(mgDf47)*, or *skn-1(zu67)* mutants worms were fed with *cgef-1*(RNAi) or *control*(RNAi). Data from a representative experiment are shown in which animals were exposed to 35°C. Replicates and statistics are presented in Supplementary Table 2. (**F**) The oxidative stress resistance phenotype (TBHP) of *cgef-1*(RNAi) requires *skn-1* function. Wild type N2 and *skn-1(zu67)* mutants fed with *cgef-1*(RNAi) or *control*(RNAi) were exposed to 7.5 mM TBHP. A representative experiment is shown. See Supplementary Table 3 for replicates and statistics.

Two transcription factors, DAF-16 and SKN-1, have been implicated in the prolonged lifespan that results from genetic inhibition of mTORC1 [16]. To test the roles of *daf-16* and *skn-1* in *cgef-1*-mediated longevity we analyzed the lifespan of *skn-1* and *daf-16* mutants upon *cgef-1*(RNAi) knockdown. We found that mutation of *daf-16* strongly suppressed the lifespan extension associated with inhibition of *cgef-1* (Figure 3A and Table 1). Inactivation of *cgef-1* by RNAi, however, still increased the lifespan in *skn-1(zu67)* mutants (Figure 3B and Table 1). A *daf-16;skn-1* double mutant completely eliminated the increase in lifespan deriving from *cgef-1*(RNAi) (Figure 3C and Table1). We conclude that DAF-16 profoundly contributes to the increases in longevity that are associated with reductions in *cgef-1* activity, while SKN-1 is not required.

Next, we tested whether the *cgef-1* phenotype of elevated stress resistance require *daf-16* and *skn-1*. We observed that the increased resistance of *cgef-1*(RNAi) to heat stress was completely abolished by mutation of *daf-16* (Figure 3D), while mutation of *skn-1* only partially prevented the thermo-tolerance (Figure 3E). Consistent with the role of *skn-1* in phase 2 detoxification, mutation of *skn-1* dramatically suppressed the increases in oxidative stress resistance seen with *cgef-1*(RNAi) (Figure 3F). These data suggest that the increases in heat stress resistance resulting from *cgef-1* knockdown are predominantly mediated by DAF-16, while SKN-1 is essential under TBHP oxidative stress conditions.

**Figure 4:**
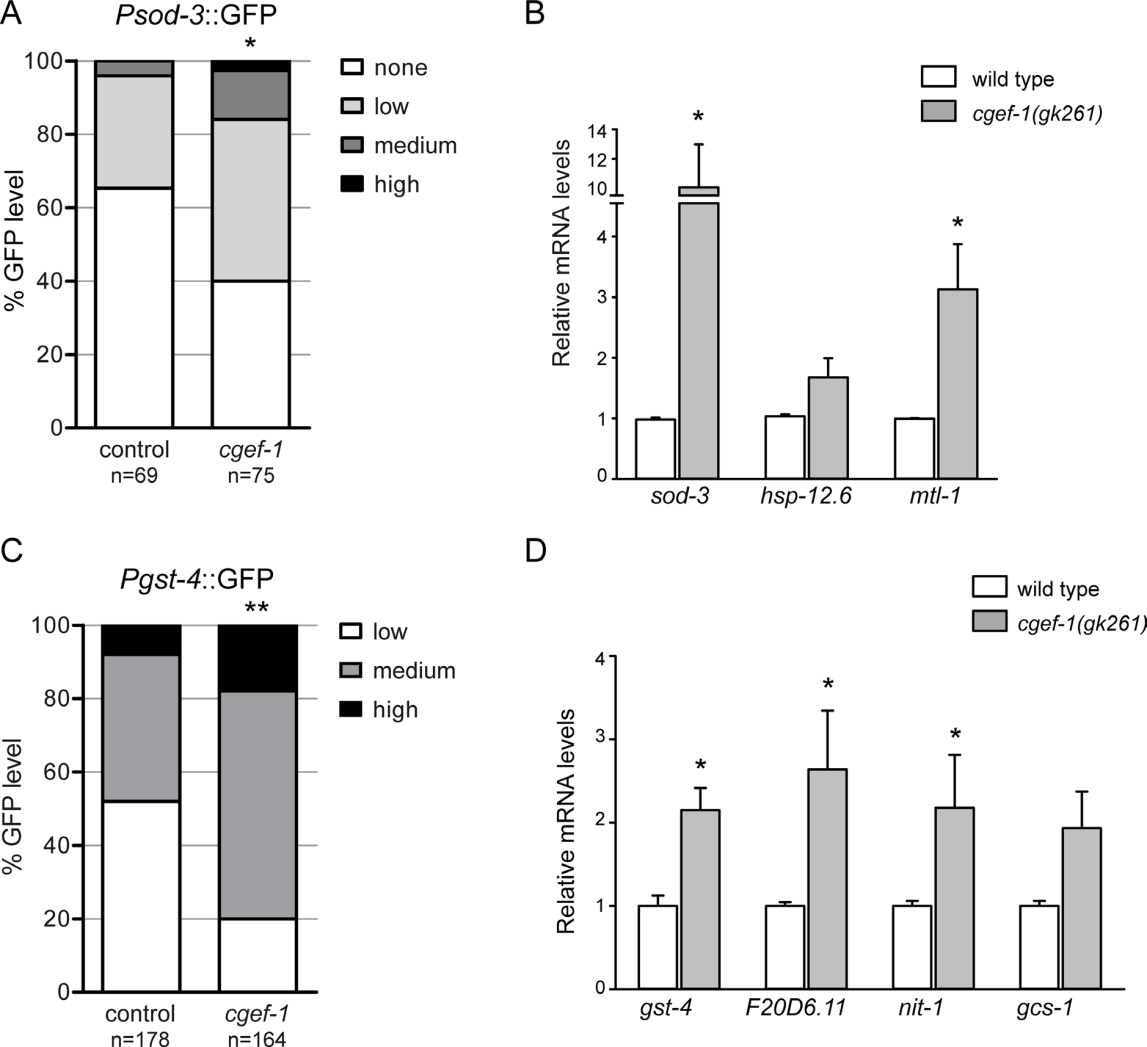
Inhibition of *cgef-1* induces DAF-16 and SKN-1-mediated transcription (**A**) Analysis of *Psod-3*::GFP expression after exposure to *cgef-1*(RNAi) or *control*(RNAi) in adult worms. Induction of *sod-3* expression was quantified as described (see Experimental Procedures). *p*-values were calculated by the Chi-square-test. ^*^p < 0.05. n, number of animals analyzed. Pooled data from three experiments. (**B**) Induction of endogenous DAF-16 target gene expression in *cgef-1(gk261)* mutants analyzed by qPCR. Data are presented as fold change compared to wild type averaged from three independent experiments, error bars represent SEM. *p*-values were derived from a Student’s *t*-test. ^*^p < 0.05. (**C**) *Pgst-4*::GFP expression is induced in response to *cgef-1*(RNAi) knockdown. Induction of the *Pgst-4*::GFP reporter was quantified as described (see Experimental Procedures). *p*-values were calculated by the Chi-square-test. ^**^p < 0.01. n, number of animals analyzed. Pooled data from three experiments. (**D**) Activation of endogenous SKN-1 target genes in *cgef-1(gk261)* mutants analyzed by qPCR. Data are mean ± SEM. *p*-values were derived from a Student’s *t*-test. ^*^p < 0.05.

We wondered whether the transcription activities of SKN-1 and DAF-16 are actually controlled by *cgef-1*. DAF-16 and SKN-1 orchestrate a well described gene expression program [28, 29]. When mTORC1 components are knocked down by RNAi, several DAF-16 and SKN-1 targets are upregulated [16]. Accordingly, we observed that the transcriptional reporter *Psod-3*::GFP, in which the promoter of the conserved DAF-16/FoxO target superoxide dismutase (*sod-3)* is fused to GFP, was activated in the intestine through *cgef-1*(RNAi) (Figure 4A). We also analyzed the effects on endogenous DAF-16 regulated gene expression in *cgef-1* mutants using quantitative PCR. We assayed mRNA production from the well-characterized DAF-16 targets *sod-3*, *mtl-1,* and *hsp-12.6* [29]. *cgef-1* mutation increased the expression of *sod-3* and *mtl-1*, but not the small heat shock gene *hsp-12.6* (Figure 4B). We conclude that, when *cgef-1* is inactivated, DAF-16 induces a protective transcriptional response.

We further investigated whether *cgef-1* also regulates the transcriptional activity of SKN-1. We examined how *cgef-1*(RNAi) affected the expression of the conserved SKN-1 target glutathione S-transferase *gst-4* using a transcriptional P*gst-4*::GFP reporter. In the intestine, P*gst-4*::GFP was expressed at low levels under normal conditions, but was strongly upregulated by inhibition of *cgef-1* through RNAi (Figure 4C). We also analyzed effects on endogenous SKN-1 regulated gene expression focusing on a set of target genes involved in various stress processes, such as *gst-4*, *gcs-1*, *nit-1,* and oxidoreductase F20D6.11 [28]. We observed that the expression of all four genes was increased in *cgef-1* mutants (Figure 4D). Taken together, these data indicate that both SKN-1 and DAF-16 induce protective gene expression when *cgef-1* is inactivated.

### Dbl interacts with human Rheb and activates mTORC1

**Figure 5:**
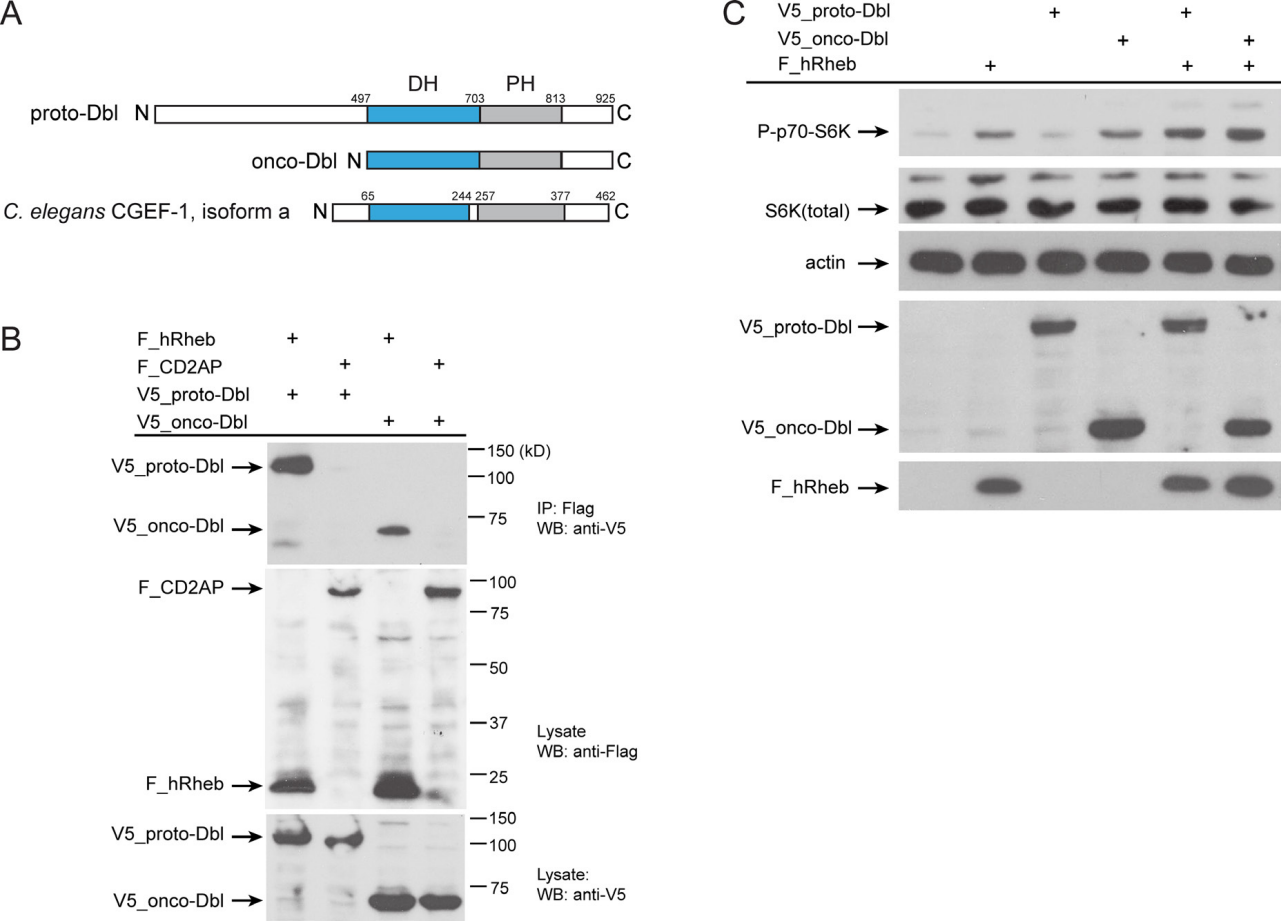
Human Dbl interacts with Rheb-1 (**A**) Schematics of the proto-Dbl, onco-Dbl and the CGEF-1 protein domain structure. The Dbl homology (DH) domain is shaded blue, the PH pleckstrin homology (PH) domain grey. Oncogenic activation of proto-Dbl occurs through truncation of the N-terminal 497 residues. The C-terminal half of Dbl includes the DH domain and PH domain which constitutes the minimum module essential for cell transformation. (**B**) Dbl proteins interact with human Rheb (hRheb) in HEK293T cells. HEK293T cells expressing V5-tagged proto-Dbl or onco-Dbl and Flag-tagged hRheb-1 or CD2AP as indicated were subjected to immunoprecipitation (IP) with anti-Flag antibody. Association of V5-Dbl with Flag-hRheb was determined by western blot (WB) analysis with anti-V5 antibody (upper panel). The control protein CD2AP failed to bind Dbl. Middle part shows expression of Flag-tagged proteins, the lower panel shows the expression of V5-tagged proto- and onco-Dbl in cell lysates. kD, kiloDalton. (**C**) Onco-Dbl but not proto-Dbl induces S6K phosphorylation in HEK293T cells. V5-tagged proto-Dbl or onco-Dbl and Flag-tagged hRheb were co-expressed in transiently transfected HEK 293T cells. Cell lysates were analyzed by immunoblotting with the indicated antibodies. Anti-actin was used to verify equivalent input of total cellular protein.

*C. elegans* CGEF-1 displays the N- to C-terminal Dbl-homology (DH) and pleckstrin-homology (PH) domain structure shared by the Dbl family of GEFs (Figure 5A) [30]. A database search identified human Dbl (also called MCF2) as potential ortholog of *C. elegans* CGEF-1. *C. elegans* CGEF-1 showed 30 % identity to the human Dbl protein. Dbl is a prototype member of GEFs that stimulate the activation of GTPases from the Rho family [31]. Mutations of the *dbl* gene leading to deregulated GEF activity and persistent stimulation of the GTPase substrate are associated with cancer. Oncogenic activation of the *dbl* proto-oncogene involves the loss of the amino-terminal half of the protein giving rise to a constitutively active GEF with potent transforming potential (Figure 5A) [32]. Given the homology and structural similarity, we analyzed whether Dbl can also interact with human Rheb (hRheb) by co-immunoprecipitation from HEK 293T cells. hRheb specifically precipitated both, the full-length proto-oncogenic form of Dbl (proto-Dbl) and the truncated oncogenic form of Dbl (onco-Dbl) (Figure 5B).

Next, we tested whether Dbl can promote mTORC1 signaling. Therefore epitope-tagged hRheb, proto-Dbl, and onco-Dbl were expressed in HEK cells and the phosphorylation level of the mTORC1 substrate S6 Kinase was evaluated. Previous studies have shown an increase in the phosphorylation of S6 Kinase when Rheb was overexpressed in HEK293 cells [3, 4]. Accordingly, we observed increased S6 kinase phosphorylation by overexpression of hRheb (Figure 5C). Intriguingly, overexpression of onco-Dbl, but not the full length proto-Dbl resulted in enhanced phosphorylation of S6 kinase (Figure 5C). Co-expression of hRheb together with onco-Dbl further increased S6K phosphorylation levels. These observations suggest that Dbl can associate with Rheb to activate the mTORC1 pathway and this interaction is conserved between *C. elegans* and humans.

## DISCUSSION

Our *C. elegans* and biochemical experiments have identified the guanine nucleotide exchange factor CGEF-1 as a novel regulator of the mTORC1 pathway. CGEF-1 directly binds to RHEB-1 and stimulates mTORC1 signaling activity to mediate stress responses and aging. Absence of *cgef-1* results in longevity similar to deficiency of mTORC1. Previous studies uncovered effects of mTORC1 inhibition that may enhance lifespan and stress tolerance including decreased protein synthesis, increased autophagy, and induction of a protective gene expression program by the transcription factors DAF-16/FoxO and SKN-1/Nrf2 [16, 19, 20, 23]. Intriguingly, we found that *cgef-1* involves these mTORC1-associated mechanisms. The finding that human Dbl also associates with Rheb and regulates well-established mTORC1 targets indicates a conserved function of CGEF-1/Dbl proteins in invertebrates and in mammals.

mTOR signaling is evolutionarily highly conserved and has a fundamental role in metabolism, cell growth, and aging [1, 2]. Genetic evidence from several model organisms established Rheb as an indispensable upstream activator of mTORC1 signaling by growth factors and amino acids [33]. Thus *C. elegans* RHEB-1 served as an attractive bait protein in our yeast-two hybrid screen for mTORC1 regulators. Thereby, we identified and verified CGEF-1 as a novel binding partner of RHEB-1 (Figure 1A). Dbl, the mammalian ortholog of *C. elegans* CGEF-1, is also associated with human Rheb (Figure 5B), indicating that the interaction is conserved across species. *cgef-1* encodes a guanine nucleotide exchange factor and has previously been involved in the activation of the GTPase CDC-42 during cell polarization and asymmetric division in the *C. elegans* embryo [24, 30]. Dbl was initially identified as an oncogene in mammalian cells causing tumorgenic growth. Dbl proteins function as guanine nucleotide exchange factors for Rho family GTPases facilitating the exchange of GDP to GTP and thus stimulating the active GTP-bound state. Dbl family proteins share a structural tandem module of DH and PH domains responsible for activation of Rho [31]. Dbl proteins activate Rho GTPases in diverse biological processes, including actin cytoskeletal dynamics, cell growth, proliferation, and survival. Aberrant function of Dbl proteins has been implicated in malignant transformation, invasion and metastasis [31].

Based on the initial finding that CGEF-1 directly binds to RHEB-1 we have been able to show that *cgef-1* affects mTORC1 signaling *in vivo*. The expression pattern of *cgef-1* in *C. elegans* substantially overlaps with *rheb-1* and *let-363*/*CeTOR* (Supplementary Figure 1B–1D). Interestingly, *cgef-1* is strongly expressed in the intestine where mTOR activity is required for adaptation to environmental changes [16]. *cgef-1* mutants and also *cgef-1* knockdown by RNAi during adulthood shared the longevity and stress phenotypes associated with loss of *rheb-1, let-363/CeTOR* kinase and *daf-15/*Raptor function. Moreover, the longevity produced by inhibition of *rheb-1* and mTORC1 was not further increased by *cgef-1* mutation (Figure 1E–1G, and Figure 2A–2B), and overexpression of *rheb-1* completely suppressed *cgef-1* associated longevity (Figure 1H). Together, our observations are consistent with a role of CGEF-1 upstream in the Rheb/mTORC1 pathway. Of note, *cgef-1* mutants exhibit only mild developmental phenotypes compared to loss of *rheb-1, let-363*/*CeTOR*, and *daf-15*/*Raptor* function (Supplementary Figure 4). Absence of *rheb-1* or mTORC1 results in arrest at the L3 larval stage [12, 13, 15] while *cgef-1* mutants display no severe embryonic lethality or larval developmental defects. The *cgef-1(gk261)* deletion destructs the DH domain essential for GEF function and therefore was previously characterized as strong loss of function or null allele [24]. However, *cgef-1(gk261)* does not completely eliminate CDC-42 activity during asymmetric cell division in the embryo indicating the presence of alternative factors [24, 30]. Indeed, a large number of GEFs is present in *C. elegans* [34] and other animals, and redundant activation by multiple GEFs may be a common theme [35]. Thus, a more complex and partially redundant GEF network might control mTORC1 activity.

We further implicated CGEF-1 in the regulation of mTORC1 and showed that suppression of *cgef-1* caused inactivation of mTORC1 downstream signaling. Active mTORC1 regulates a set of well-characterized targets including S6 kinase and the translational repressor 4E-BP1 to stimulate protein synthesis, and inhibits catabolic processes like autophagy [10]. Knockdown of *cgef-1* in *C. elegans,* similarly to inhibition of *rheb-1* and mTORC1 gene function, reduced the phosphorylation of human 4E-BP1 (Figure 2C). In mammals, 4E-BP1 is phosphorylated in response to various signals resulting in its dissociation from the translation initiation factor 4E (eIF4E) and activation of cap-dependent mRNA translation. Consistent with mTORC1 signaling being downregulated by loss of *cgef-1* function, we also observed increased LGG-1 positive autophagic vesicles in the intestine (Figure 2D, 2E). Together, our data show that CGEF-1 engages mTORC1-associated mechanisms and therefore we link CGEF-1 with RHEB-1 as regulator of mTORC1 signaling.

Studies in several organisms demonstrate that autophagy and translational processes are central effector mechanisms of mTORC1 on aging [19, 23, 36]. Moreover, mTORC1 signaling influences longevity by opposing the transcription factors DAF-16/FoxO and SKN-1/Nrf2 in *C. elegans* [14, 16]. When the mTORC1 pathway is inhibited, DAF-16 and SKN-1 activate a protective gene expression response, and increase stress resistance and longevity. We observed that mutation of *daf-16* completely suppressed the extended lifespan of *cgef-1*(RNAi) (Figure 3A). *cgef-1*(RNAi), however, increased lifespan modestly, but reproducibly, in wild-type animals and *skn-1* mutants (Figure 3B). These observations indicate that longevity upon loss of *cgef-1* involves DAF-16, but not SKN-1. Along that line, DAF-16 was also critical for thermo-tolerance associated with inactivation of *cgef-1* while SKN-1 played a less important role (Figure 3D, E). Notably, it was shown in a previous study that delays in aging resulting from mTORC1 inhibition by knockdown of the RAG GTPases *ragc-1* and *raga-1* required both DAF-16 and SKN-1 [16]. Perhaps DAF-16 and SKN-1 are affected differently by these regimens. The enhanced oxidative stress resistance derived from *cgef-1*(RNAi), however was completely abolished in *skn-1* mutants suggesting that SKN-1 is the central player for phase 2 detoxification and adaptation to oxidative stress (Figure 3F). Inactivation of *cgef-1* resulted in upregulation of a set of genes (Figure 4) for which the expression has been shown to strongly depend on DAF-16 and SKN-1 [29, 37]. Together, these findings support the notion that the transcriptional responses of DAF-16 and SKN-1 are critical for *cgef-1* mediated longevity and protection from environmental stress, as was observed previously for genetic mTORC1 inhibition [16].

Our observations would provide a model whereby CGEF-1 stimulates mTORC1 signaling to regulate key substrates for protein synthesis, autophagy and longevity in *C. elegans*. How could CGEF-1 promote mTORC1 signaling? It is well established that Rheb plays a crucial role in the activation of mTORC1 upon growth factor or amino acid stimulation [33]. One might speculate that CGEF-1 straightly activates RHEB-1 to promote mTORC1 signaling. Rheb is negatively controlled by the TSC complex that functions as GAP promoting conversion of active Rheb to the inactive form. This leads to decreased mTORC1 activity [5, 38]. No obvious homologs of the TSC complex have been identified in the nematode. Interestingly, it was described that RalGAP (HGAP-1 and HGAP-2) shares similarity with TSC and loss of *hgap-1/hgap-2* phenocopied mTORC1 activation and decreased lifespan in *C. elegans*, consistent with a TSC-like function [39]. The role of GEF molecules that counter the activity of the TSC in the control of Rheb remains so far ill-defined. Studies in *Drosophila* identified the translationally controlled tumor protein (TCTP) functioning as GEF to activate Rheb and implicated TCTP in the mTORC1 pathway [40]. Yet, following studies reported that TCTP does not affect mTORC1 signaling in mammals [41, 42]. Hence, so far, no positive regulator of Rheb activity has been identified in the mammalian system. Our study now proposes CGEF-1 as a new activator of RHEB-1/mTORC1 signaling. Genetic analyses establish that *cgef-1* is epistatic to *rheb-1* and functions upstream of mTORC1 in the control of aging. Biochemical experiments show that CGEF-1 and RHEB-1 form a complex together (Figure 1A). Unfortunately, we have not detected direct *in vitro* GEF activity towards RHEB-1 due to technical problems of purifying functional CGEF-1 protein. Notably, CGEF-1 has been described as GEF and activator of the Rho GTPase CDC-42 [24, 30]. Yet, multiple lines of evidence indicate that some GEFs can target and activate multiple GTPases [reviewed in 31]. An alternative model is that the ability of CGEF-1 to regulate mTORC1 activity is rather indirect. CGEF-1 might facilitate RHEB-1/mTORC1 complex formation and thereby promote mTORC1 signaling.

Strikingly, the regulation of mTORC1 signaling by CGEF-1/Dbl proteins appears to be conserved in evolution. First, we observed that human Dbl can engage complexes with Rheb (Figure 5B). The oncogenic form of Dbl bearing the DH-PH domains that promote nucleotide exchange *in vivo* was sufficient to bind hRheb (Figure 5B). Next, overexpression of onco-Dbl, just as Rheb in cell culture, strongly increased the phosphorylation level of S6K (Figure 5C) supporting the hypothesis that Dbl stimulates mTORC1 activity. It will be of great importance to elucidate how Dbl/CGEF-1 influences mTORC1-mediated mechanisms and longevity across species.

## MATERIALS AND METHODS

### *C. elegans* growth conditions

Unless otherwise indicated *C. elegans* were cultured at 20°C on standard NGM plates seeded with *E. coli* OP50. Nematode strains used in this study are described in Supplementary Table 4.

### RNA interference

RNAi experiments were carried out as described [43]. HT115 bacteria transformed with RNAi clones or empty vector pL4440 were grown over night in 12.5 µg/ml tetracycline and 50 µg/ml ampicillin. The following day, cultures were diluted 1:10 and grown to an OD600 of 0.8-1.0 and induced with 0.7 mM IPTG. This culture was used to seed NGM plates containing tetracycline, ampicillin, and 1 mM IPTG. RNAi plasmids for knockdown of *cgef-1* and *rheb-1* were derived from the ORF-RNAi library. *skn-1*(RNAi) plasmid was obtained from K. Blackwell [37] and *let-363*(RNAi) plasmid was previously described [13]. For *daf-15*(RNAi) partial cDNA was cloned into pPD129.36. All clones were confirmed by sequencing.

### Lifespan analysis

Lifespan assays were performed at 20°C and measured from L4. At L4 stage, worms were transferred to NGM plates containing 50 µM FUdR or RNAi plates with FUdR. Animals were scored daily for surviving. Animals that crawled off the plate, ruptured, or showed internally hatched larvae were censored. Data were analyzed using a log-rank test with Holm-Sidak method performed with SigmaStat 3.5.

### Plasmids and transgenic strains

We generated a *rheb-1::gfp* translational fusion by inserting a SalI/BamHI fragment containing the complete genomic *rheb-1* locus and 2.8 kb upstream regulatory sequence into pPD95.75 (A. Fire, Carnegie Institute of Washington, Baltimore, MD). To generate animals overexpressing *rheb-1,* the *rheb-1::gfp* plasmid (ID8424) was injected at 10 ng/μL into *unc-119(ed4)* mutants along with *unc-119* wild-type rescuing plasmid (50 ng/μL).

For the analysis of 4E-BP phosphorylation in *C. elegans,* Flag-tagged human 4E-BP1 cDNA was cloned under control of 2.5 kb *ges-1* promoter into pPD30.38 (ID8132). Transgenic strains expressing h4E-BP1 were created by injection of the P*ges-1::*Flag_h4E-BP1 plasmid at 10 ng/µl along with 20 ng/µl pRF4 (*rol-6*) in N2. Extrachromosomal arrays of Flag_h4E-BP1 were integrated into the genome by γ-irradiation. The integrated lines were backcrossed at least four times with wild type N2.

We generated *cgef-1::gfp* translational fusion by inserting a 1386 bp fragment of *cgef-1a* cDNA under control of 2280 bp *ges-1* promotor region into pPD95.75. *Pges-1::cgef-1::GFP* plasmid (ID9096) was injected at 20 ng/µl into wild-type N2 animals along with the coinjection marker 20 ng/µl pRF4 (*rol-6*) to generate animals expressing *cgef-1::*GFP.

### Analysis of brood size

Animals were synchronized by timed egg laying on OP50 or RNAi and grown to the L4 stage at 20°C. To assess the brood size, individual animals were transferred every other day onto plates until egg-laying ceased and their total number of progeny was counted.

### Analysis of body size

Animals were raised at 20°C and fed with RNAi for two generation. F1 generation was synchronized by egg laying and after 72 hours, worms were anaesthetized with Tetramisolhydrochloride (2mM) and images were taken at 5x magnification. To measure the body size, the perimeter was traced and the area was calculated using the software application AxioVision4 (Zeiss).

### Microscopy and transgenic reporter scoring

Light microscopy was performed using a Zeiss Axioplan 2-microscope equipped with Nomarski differential interference contrast (DIC), an AxioCam-camera and the AxioVision-Software Rel.4.8. GFP was detected using appropriate EGFP-filter sets (480/20 nm excitation, 510/20 nm emission). Images were processed and figures compiled with Adobe Illustrator CS5.

Intestinal *Pgst-4::*GFP expression was scored as essentially published [16, 21]. Worms were raised on *cgef-1*(RNAi) or *control*(RNAi) until L4 stage, then placed on slides on 2% agarose pads and stunned by levamisole. “High” refers to high GFP levels anteriorly and throughout most of the intestine, “medium” refers to intense GFP levels only anteriorly or posteriorly, and “low” refers to no or only barely visible GFP expression. Chi-square tests were performed with SigmaStat 3.5.

Intestinal *Psod-3::*GFP expression was scored based on the intensity of GFP expression as described [44]. Worms were raised on *cgef-1* (RNAi) or *control* (RNAi) until young adult. “High” refers to bright GFP expression throughout most of the intestine, “medium” refers to GFP expression only anteriorly or posteriorly, “low” refers to weak visible GFP expression, and “no” indicates barely detectable GFP expression. Chi-square tests were performed with SigmaStat 3.5.

GFP intensity of animals expressing *cgef-1*::GFP and *rheb-1*::GFP was analyzed with a Zeiss Axiovert 200 M microscope and AxioCam camera. GFP fluorescence intensity of the images was measured with ZenBlue2011. Background intensity was subtracted. Fluorescence intensity of the wild type was set to 100%.

### Analysis of autophagy in *C. elegans*

The level of autophagy in intestinal cells was assessed using a *Pnhx-2*::*mCherry::lgg-1* translational reporter characterized previously [27]. Animals were raised at 20°C and fed with RNAi until L4. L4 larvae were then scored for mCherry-positive puncta using an Axioplan 2 microscope at high magnification (630x). Foci were counted based on the intensity of mCherry expression in the proximal region of the intestine as described [27, 45]. Three independent biological samples were analyzed for autophagic events. ANOVA test was performed with SigmaStat 3.5.

### Heat and oxidative stress resistance assays

To assess heat resistance, L4 stage worms were fed with RNAi bacteria for four days at 20°C. Worms were then shifted to 35°C and tested for survival. For oxidative stress resistance, worms that had been treated as described above were transferred to NGM plates that contained 7.5mM *tert*-Butyl hydroperoxide (TBHP) (Sigma-Aldrich) and were seeded with OP50. Animals were scored for surviving and worms that did not respond to gentle prodding with a platinum wire were scored as dead. Worms that crawled off the plate, ruptured, or died from internal hatching of progeny were excluded from the analysis. Survival plots and *p*-values (log-rank) were determined using SigmaStat software.

### RNA Isolation and quantitative PCR

Total RNA was isolated from four day adult worms using TRI Reagent (Sigma-Aldrich) and a RNA clean and concentrator kit (Zymo Research Corp.). DNase treatment was performed using the on-column DNase digestion (Quiagen). To generate cDNA 1 µg of RNA was reverse transcribed with oligo-dT primer and the Superscript III Kit (Invitrogen).

Quantitative PCR reactions were performed with Roche LightCycler 480 using Takyon No Rox SYBR MasterMix dTTP Blue (Eurogentec). qPCR reactions were performed in at least three independent samples in triplicates. The quantification of changes in mRNA expression levels was based on the 2^–ΔCt^ method [46]. Results were normalized against endogenous *cdc-42* and *Y45F10D.4* expression. Significant differences were assessed by Student’s unpaired *t*-test. Primer sequences are listed in Supplementary Table 6.

### Analysis of h4E-BP phosphorylation in *C. elegans*

Worms carrying the *Is[Pges-1::Flag-h4E-BP1]* transgene (ENH441) were collected at young adult stage in IP lysis buffer containing 40 mM HEPES (pH7.5), 120 mM NaCl, 1 mM EDTA and 0.3 % CHAPS supplemented with 1 mM PMSF and protease inhibitor cocktail (Sigma), and dissolved by sonication (10-s sonication with a 10-s break, 10 cycles) and incubation with constant rotation for two hours at 4°C to fully lyse the worms. FLAG-tagged h4E-BP1 was immunoprecipitated with anti-FLAG M2 affinity beads (Sigma). Immunoprecipitates were washed with CHAPS-free IP lysis buffer three times at 4°C, and eluted by boiling with 2× SDS sample buffer at 95°C for 3 min. Immunoprecipitates were subjected to subsequent western blot analysis using anti-FLAG antibody (Sigma) and anti-phospho-4E-BP1 antibody (Cell Signaling).

### Yeast split-ubiquitin two-hybrid interaction screen

The RHEB-1 protein interaction screen was performed using the yeast two-hybrid (Y2H) split-ubiquitin system as described before [47]. As bait the coding sequence of *C. elegans rheb-1* was fused to the C-terminus of ubiquitin (Cub) in pPcup1-ubc9-CRU and used for transformation of the yeast strain *JD53*. A *C. elegans* Nub I-split ubiquitin cDNA library was used as prey. Positive clones were identified by sequencing and BLAST with the *C. elegans* genome.

### Cell culture, immunoprecipitation, and immunoblotting

HEK293T cells were grown in DMEM with 10% FBS. Cells were transfected with plasmid DNA applying the calcium phosphate method and incubated for 24h. Cells were lysed in IP buffer (1% Triton-X 100, 20 mM Tris pH 7.5, 50 mM NaCl, 50 mM NaF, 15 mM Na4P2O7, 0.1 mM EDTA pH 8.0) supplemented with protease inhibitors. After centrifugation (43,000 rpm, 30 min, 4°C) the supernatants containing equal amounts of total protein were incubated with anti-FLAG M2 affinity beads (Sigma) for at least 1 h at 4°C. The precipitates were washed extensively with IP buffer and subjected to SDS-PAGE and immunoblotting analysis with anti-Flag and anti-V5 antibodies (Sigma).

For the analysis of S6K phosphorylation HEK293T cells were transfected with epitope-tagged Rheb and Dbl protein. 24h after transfection, cells were starved 1h in MEM, Hank’s Balanced Salts (from life technologies) and then lysed in IP buffer. Lysates were centrifuged (43,000 rpm, 30 min, 4°C). Equal amounts of protein samples were analyzed by immunoblotting. Anti-p70-S6K and anti-phospho-p70-S6K antibodies (Thr389) were obtained from Cell signaling, anti-actin antibodies from Sigma.

Expression constructs are described in detail in the Supplementary Table 5.

## SUPPLEMENTARY MATERIALS FIGURES AND TABLES




