# Supplementary material for: CGEF-1 regulates mTORC1 signaling during adult longevity and stress response in C. elegans
Source: Oncotarget. 2018 Jan 6;9(11):9581–95. doi: 10.18632/oncotarget.24039 (PMC5839386; doi:10.18632/oncotarget.24039)
Supplement: Supplementary file 1 [file oncotarget-09-9581-s001.pdf]

CGEF-1 regulates mTORC1 signaling during adult longevity and stress response in C. elegans

SUPPLEMENTARY MATERIALS

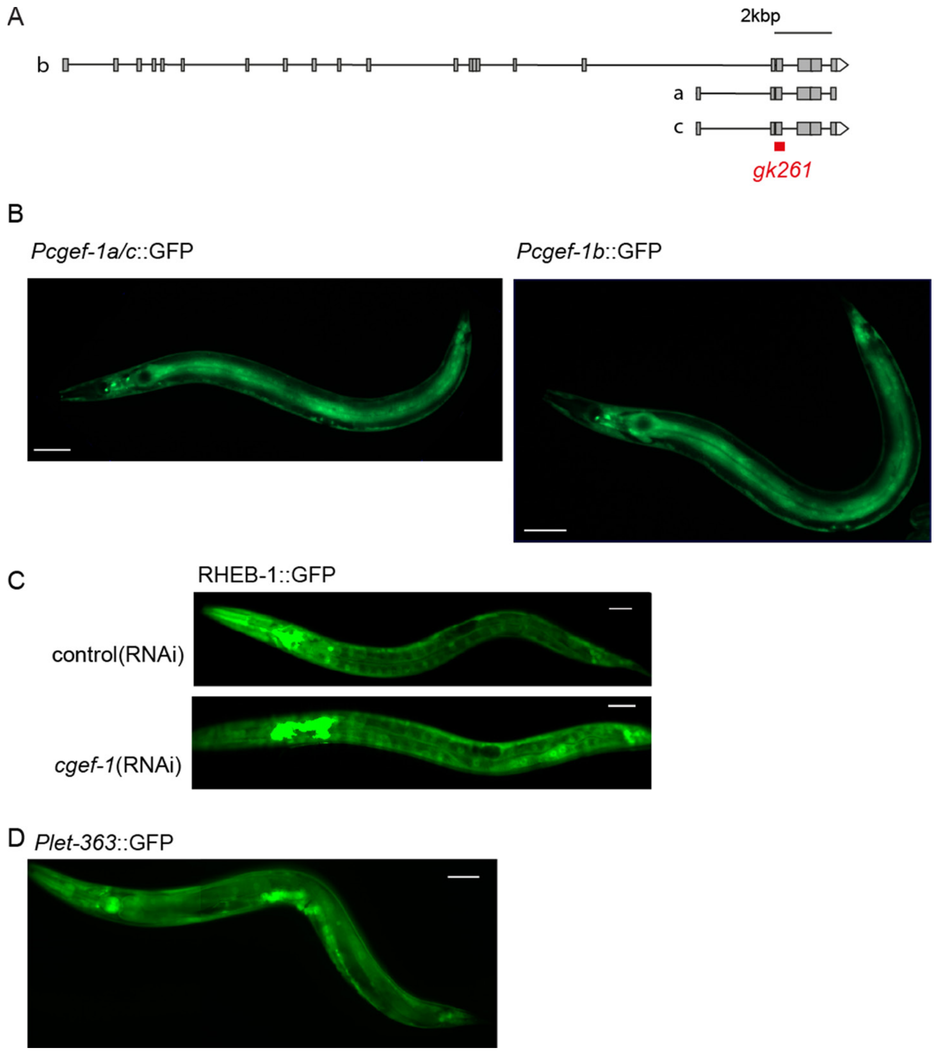

Supplementary Figure 1: Expression of *cgef-1* overlaps with *rheb-1* and *let-363/CeTOR.*(A) Genomic organization of *cgef-1* isoforms and location of the deletion in gk261 mutants. Coding regions are indicated by boxes, and introns are represented as lines. *cgef-1(gk261)* mutants bear a deletion of 318 bp including exon3 and producing a frameshift with early translational stop [24]. This deletion is expected to disrupt the GEF function including the DH and PH domain. (**B**) Expression pattern of *Pcgef-1a/c*::GFP (NK775) and *Pcgef-1b::*GFP (NK774) in *C. elegans*. Tissues expressing *cgef-1* were determined with transgenes expressing GFP under the control of 5' cis-regulatory sequences. NK774 bears 4054 bp upstream regulatory sequence of *cgef-1b* fused to GFP. NK775 contains 3063 bp cgef-1a/c promoter sequence [48]. *cgef-1a/c*::GFP (left panel) and *cgef-1b*::GFP (right panel) are broadly expressed the pharynx, the intestine, the vulva area, the majority of head and tail neurons, and the hypodermis. Bar represents 50 µm. (**C**) RHEB-1::GFP is broadly expressed in many tissues including head and tail neurons, hypodermis, vulva muscles, and intestine throughout all stages of post-embryonic development and adulthood (upper panel). Knockdown of *cgef-1* by RNAi does not apparently alter the expression of RHEB-1::GFP (lower panel). To determine the expression of RHEB-1, transgenic animals carrying a rheb-1 translational fusion to GFP (ENH149) were fed with *cgef-1*(RNAi) or *control*(RNAi) for two generations and then analyzed by Axioplan 2-microscope. Scale bar represents 20 µm. Notably, the effectiveness of *cgef-1*(RNAi) was validated in Supplementary Figure 3A–3C. (**D**) Expression pattern of *Plet-363*::GFP. *let-363*/CeTOR is broadly expressed in the pharynx, the intestine, the majority of head and tail neurons, muscles, vulva, and the hypodermis. Bar represents 50 µm.

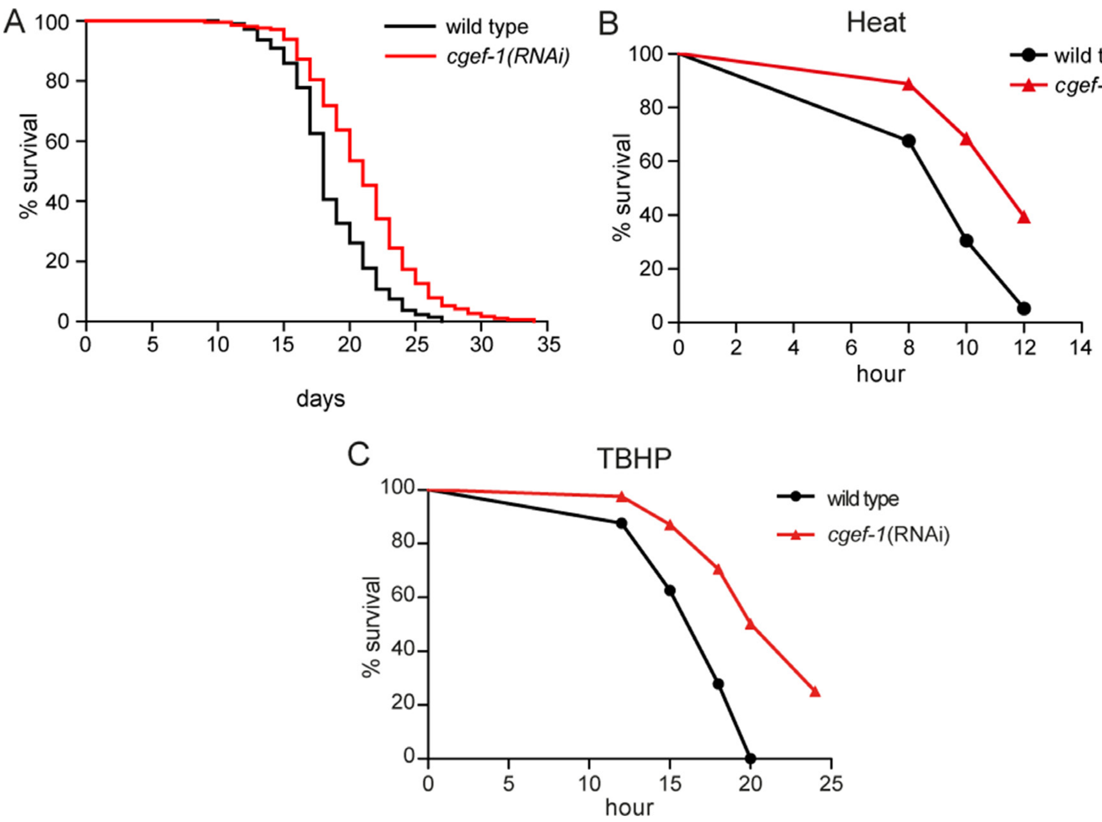

Supplementary Figure 2: Inhibition of *cgef-1* by RNAi increases lifespan and stress tolerance.(**A**) Lifespan analysis of wild type N2 fed with *cgef*-1(RNAi) or *control*(RNAi) starting from L4. Survival plot shows combined data from three experiments. See also Table 1 for corresponding data and statistics. (**B**) *cgef-1*(RNAi) knockdown increases heat resistance. Data from a representative experiment are shown. Results of replicates and statistics are presented in Supplementary Table 2. (**C**) Inhibition of *cgef-1* by RNAi increases resistance to oxidative stress. A representative experiment is shown in which animals were exposed to 7.5 mM TBHP. See also Supplementary Table 3 for results of replicates and statistics.

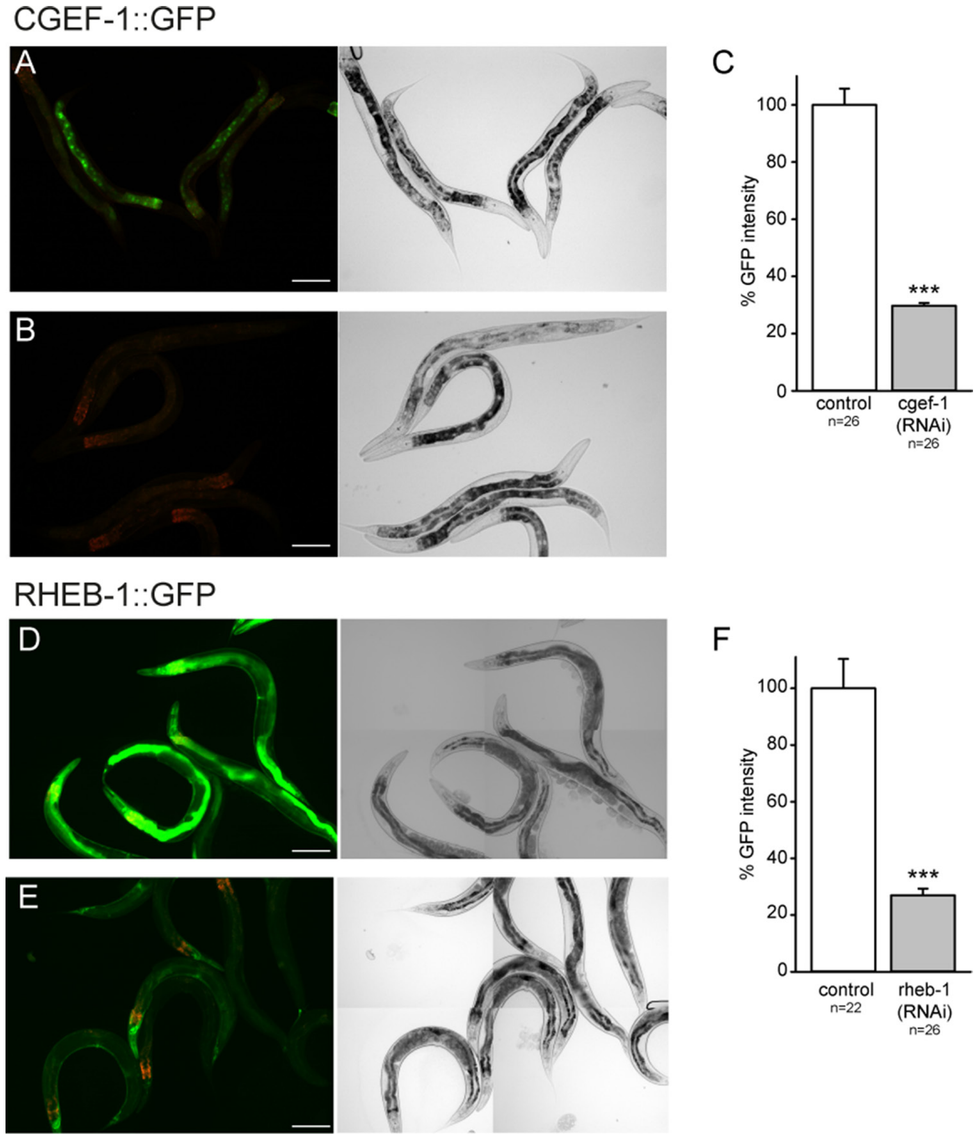

Supplementary Figure 3: Effectiveness of *cgef-1* and *rheb-1* knockdown by RNAi. (**A**–**C**) *cgef-1*(RNAi) effectively eliminates *cgef-1*::GFP expression. Transgenic animals carrying a *cgef-1* translational fusion to GFP (ENH557) were fed with *control*(RNAi) (A) or *cgef-1*(RNAi) (B) and then GFP fluorescence was analyzed by Axioplan 2 microscope. Representative GFP fluorescence (left panel) and Normarski images (right panel). Scale bar represents 100 µm.(C) Quantification of CGEF-1::GFP fluorescence intensity in young adult animals. p-values were derived from a Student's t-test. $^{***}p < 0.001$. n, number of animals analyzed. Pooled data from two experiments.(D-F) To determine the effectiveness of the *rheb-1*(RNAi), transgenic animals carrying a *rheb-1* translational fusion to GFP (ENH149) were fed with *control*(RNAi) (**D**) or rheb-1(RNAi) (**E**) and then GFP fluorescence was analyzed. Representative images of rheb-1::GFP (left panel) and corresponding Nomarski-view (right panel). *rheb-1*::GFP expression was strongly reduced after feeding of *rheb-1*(RNAi). Scale bar represents 100 µm.(**F**) Quantification of RHEB-1::GFP fluorescence intensity in young adult animals. *p*-values were derived from a Student's *t*-test. $^{***}p < 0.001$. n, number of animals analyzed. Pooled data from two experiments.

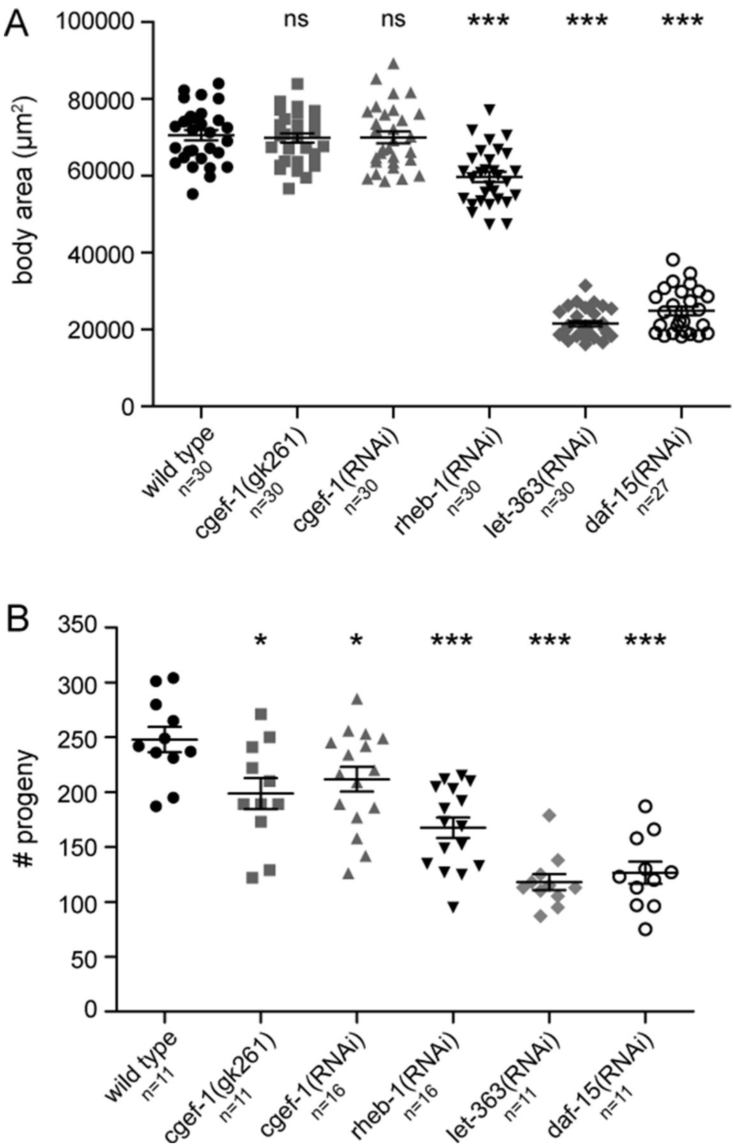

Supplementary Figure 4: Phenotypes of loss of *cgef-1* function. (**A**) Quantification of the body size. Wild-type N2 animals were fed with indicated RNAi for two generations at 20°C. (**B**) Quantification of the number of progeny. Wild-type N2 worms were fed with indicated RNAi starting from L1 and their total number of progeny was counted. Each point in the scatter plots in A and B represents the analysis from one animal. Bars indicate mean ± SEM. Data pooled from two experiments. N, number of animals analyzed. p-values were calculated by Student's *t*-test (mutant strain or RNAi knockdown relative to wild type), $^{*}p < 0.05$, $^{***}p < 0.001$. ns, not significant.

```
hDbL NM_005369     KTIQLKLENIRSIFENQQAGFRNLADKHVRPIQFVVP-------TPENLV   443
C. elegans CGEF-1  -------------------------------MKRTLPRFLSFRRSKRRLI    19

hDbl NM_005369     TS-GTPF--FSSKQGKKTWRQNQSNLKIEVVPDCQEKRSSGPSSSLDNGN   490
C. elegans CGEF-1  TSVSAPLIVMNYHDG------HQYELDSKDVPSCSGE--STPAGTLSPRR    61

                                         DH
hDbl NM_005369     SLDVLKNHVLNELIQTERVYVRELYTVLLGYRAEMDNPEMFDLMPPLLRN   540
C. elegans CGEF-1  ETREVTN-AFDEMIATEISYVADLKDIIIHYLEPFEATENQNSLPDTLRG   110

                                         DH
hDbl NM_005369     KKDILFGNMAEIYEFHNDIFLSSLENCAHAPERVGPCFLERKDDFQMYAK   590
C. elegans CGEF-1  KPDCLFGNVRELYKFHHRTVLEDLVAARSTAEMCRVLMQHRNQIYVTYRT   160

                                         DH
hDbl NM_005369     YCQNKPRSETIWRKYSECAFFQECQRKLKHRLRLDSYLLKPVQRITKYQL   640
C. elegans CGEF-1  YCQIHGSNQKVRDSVKNHPFFKECQRNANHNMDMSSYLLKPIQRIMKYQL   210

                                         DH
hDbl NM_005369     LLKELLKYSKDCEGSALLKKAL--DAMLDLLKSVNDSMHQIAINGYIGNL   688
C. elegans CGEF-1  LLGNIM---DDCPADVRDEVAMTRDSMVELLNQIDASMQQLHISGYNGDL   257

                           DH                PH
hDbl NM_005369     NELGKMIMQGGFSVWIGHKKGATKMKDLARFKPMQRHLFLYEKAIVFCKR   738
C. elegans CGEF-1  KSLGLLRLQTECDVFTYNRKKKAKLS-----RAQKRFIFFFDGAVMFCKK   302

                                         PH
hDbl NM_005369     RVES-GEGSDRYPSYSFKH--CWKMDEVGITEYVKGDNRKFEIWYGEKEE   785
C. elegans CGEF-1  RVSNPGTTLNSEPEY-FEHKFCIPIISLGYDTSSRTGASRFEVWDDAKTD   351

                                         PH
hDbl NM_005369     VYIVQASNVDVKMTWLKEIRNILLKQQELLTVKKRKQQDQLTERDKFQIS   835
C. elegans CGEF-1  AYVIETIDQTARTKWIQRLG------------KSETAQDACLE-------   382

hDbl NM_005369     LQQNDEKQQGAFISTEETELEHTSTVVEVCEAIASVQAEANTVWTEASQS   885
C. elegans CGEF-1  ----NRQRPKSWASTVSNESSCSSSTRESDSTDSTMDTNGNTQTTQV---   425

hDbl NM_005369     AEISEEPAEWSSNYFYPTYDENEEENRPLMRPV------SEMALLY--     925
C. elegans CGEF-1  ----DVP-------YSPTLDSSIDLSMDTTTPLRTSEINNEVELVDSC     462
```

Supplementary Figure 5: CGEF-1 is evolutionarily conserved. Alignment of the human Dbl protein and the C. elegans CGEF-1 protein, isoform a (Needleman-Wunsch algorithm). Yellow shading indicates conserved amino acids; the localization of the DH and PH domain within the human protein are indicated.

Supplementary Table 1: Individual lifespan analyses. See_Supplementary_Table 1

Supplementary Table 2: Heat experiments

Strain	RNAi	Mean survival (hours ± SEM)	Median	25 %ile	*P* Value N2[a]	*P* Value	T	N D	C	Figures
N2		8.1 ± 0.1	8	9			80	79	1	1C
cgef-1(gk261)		9.4 ± 0.2	9	10	< 0.001		80	79	1	1C
N2		8.8 ± 0.1	9	10			80	68	0	
cgef-1(gk261)		9.5 ± 0.1	10	-	< 0.0001		80	41	0	
N2		8.9 ± 0.1	9	10			99	93	1	
cgef-1(gk261)		9.6 ± 0.1	10	-	< 0.0001		97	54	1	
N2		11.1 ± 0.2	12	12			81	81	0	
cgef-1(gk261)		12.2 ± 0.2	12	14	< 0.001		77	74	3	
N2	control	9.9 ± 0.2	10	12			80	73	3	S2B
N2	cgef-1	11.1 ± 0.2	12	-	< 0.0001		80	48	1	S2B
N2	control	10.8 ± 0.2	12	12			96	92	4	1F, 3D
N2	rheb-1	11.7 ± 0.3	12	14	< 0.001		98	77	12	1F
cgef-1(gk261)	control	12.5 ± 0.3	12	14	< 0.0001		81	79	2	1F
cgef-1(gk261)	rheb-1	11.8 ± 0.3	12	14	< 0.0001	ns[b]	100	87	2	1F
N2	cgef-1	12.5 ± 0.3	12	14	< 0.0001		93	84	9	3D
daf-16(mgDf47)	control	10.1 ± 0.2	10	12	< 0.001		93	93	0	3D
daf-16(mgDf47)	cgef-1	9.9 ± 0.2	10	12	< 0.001	ns[c]	94	81	13	3D
N2	control	13.5 ± 0.4	14	16			90	53	37	
N2	rheb-1	14.4 ± 0.4	14	18	0.038		92	46	41	
cgef-1(gk261)	control	15.4 ± 0.3	16	18	< 0.0001		87	38	41	
cgef-1(gk261)	rheb-1	14.5 ± 0.4	14	16	0.054	ns[b]	79	20	58	
N2	cgef-1	15.1 ± 0.3	16	16	< 0.001		91	47	38	
daf-16(mgDf47)	control	12.1 ± 0.3	12	14	<0.01		94	49	45	
daf-16(mgDf47)	cgef-1	12.3 ± 0.3	12	14	0.020	ns[c]	95	45	50	
N2	control	11.0 ± 0.3	12	12			71	71	0	3E
N2	cgef-1	13.0 ± 0.4	14	16	< 0.0001		78	74	4	3E
skn-1(zu67)	control	7.7 ± 0.3	8	10	< 0.0001		86	86	0	3E
skn-1(zu67)	cgef-1	9.1 ± 0.3	10	12	< 0.0001	< 0.001[d]	64	60	4	3E
N2	control	10.5 ± 0.3	10	12			87	86	1	
N2	cgef-1	12.7 ± 0.3	12	14	< 0.0001		87	78	9	
skn-1(zu67)	control	6.7 ± 0.3	6	8	< 0.0001		67	67	0	
skn-1(zu67)	cgef-1	8.2 ± 0.4	8	10	< 0.0001	< 0.01[d]	66	61	5	

Wild-type N2 worms, c*gef*-1(*gk*261), skn-1(*zu*67), and daf-16(*mgDf*47) mutants were fed with OP50 or individual RNAi and exposed to 35°C at four days of adulthood.
D represents total number of animals dying of old age versus those in total experiment (T). C indicates censored events (i.e. worms that crawled off the plate, ruptured or died from internal hatching). SEM = standard error of the mean. ns = not significant.
p-values (log-rank test) refer to the following control experiments:
[a] N2 wild type,
[b] cgef-1(gk261);control(RNAi),
[c] daf-16(mgDf47);control(RNAi),
[d]skn-1(zu67);control(RNAi).

Supplementary Table 3: TBHP experiments

Strains	RNAi	Mean survival (hours ± SEM)	Median	25 %ile	*P* Value N2[a]	*P* Value	T	N D	C	Fig.
N2		21.1 ± 0.3	20	24			80	76	4	1D
cgef-1(gk261)		22.7 ± 0.3	24	-	< 0.0001		80	39	4	1D
N2		16.4 ± 0.2	16	18			80	46	30	
cgef-1(gk261)		17.2 ± 0.1	18	18	< 0.01		80	49	18	
N2	control	17.1 ± 0.3	18	20			80	73	7	S2C
N2	cgef-1	21.0 ± 0.4	24	-	<0.0001		80	49	13	S2C
N2	control	15.4 ± 0.3	16	18			80	75	5	1G
N2	cgef-1	18.8 ± 0.3	-	-	< 0.0001		80	26	3	
N2	rheb-1	17.3 ± 0.4	-	-	< 0.0001		76	28	26	1G
cgef-1(gk261)	control	17.3 ± 0.4	20	-	< 0.0001		80	41	9	1G
cgef-1(gk261)	rheb	16.6 ± 0.5	18	-	< 0.01	ns[b]	76	32	13	1G
N2	control	19.1 ± 0.5	20	24			80	73	7	
cgef-1(gk261)	control	21.0 ± 0.4	24	-	< 0.0001		75	43	20	
N2	rheb-1	21.6 ± 0.4	24	-	< 0.0001		80	42	15	
cgef-1(gk261)	rheb-1	20.8 ± 0.5	24	-	< 0.0001	ns[b]; ns[d]	76	36	8	
N2	control	8.5 ± 0.4	8	10			52	47	5	3F
N2	cgef-1	11.8 ± 0.7	12	14	< 0.0001		50	47	3	3F
skn-1(zu67)	control	4.8 ± 0.2	4	6	< 0.0001		57	57	0	3F
skn-1(zu67)	cgef-1	5.0 ± 0.2	4	6	< 0.0001	ns[c]	58	57	1	3F
N2	control	12.4 ± 0.5	12	16			81	77	4	
N2	cgef-1	19.0 ± 0.7	20	22	< 0.0001		79	73	6	
skn-1(zu67)	control	5.8 ± 0.3	6	8	< 0.0001		67	67	0	
skn-1(zu67)	cgef-1	5.8 ± 0.3	6	8	< 0.0001	ns[c]	65	64	1	

Wild-type N2 worms, c*gef*-1(*gk*261), and skn-1(*zu*67) mutants were fed with OP50 or individual RNAi and exposed to 7.5 mM TBHP at four days of adulthood.

D represents total number of animals dying of old age versus those in total experiment (T). C indicates censored events (i.e. worms that crawled off the plate, ruptured or died from internal hatching). SEM = standard error of the mean. ns = not significant.

p-values (log-rank test) refer to the following control experiments:

[a] N2 wild type,

[b] cgef-1(gk261);control(RNAi),

[c] skn-1(zu67);control(RNAi),

[d] rheb-1(RNAi).

Supplementary Table 4: *C. elegans* strains used in this study

Number	Genetic background	Transgene	Reference
	N2 Bristol		
ENH378	cgef-1(gk261) X		(Kumfer et al. 2010)
EU1	skn-1(zu67)/nT1[unc-?(n754);let-?] (IV;V)		(Bowerman et al. 1992)
	daf-16(mgDf47) I		
LD1263	daf-16(mgDf47) I;skn-1(zu67)/nT1 (IV;V)		
VK1093	N2	Ex[Pnhx-2::*mCherry::lgg-1]*	(Gosai et al. 2010)
NK774	N2	qxEx116[cgef-1b::GFP]	(Ziel et al. 2009)
NK775	N2	qxEx117[cgef-1a/c::GFP]	(Ziel et al. 2009)
ENH149	unc-119(ed3) III	Ex[Prheb-1::rheb-1::GFP; unc-119(+]	This study
ENH441	N2	Is[Pges-1::Flag-h4EBP1;rol-6]	This study
ENH116	N2	Ex[Plet-363::GFP]	
ENH557	N2	Ex[ges-1P::cgef-1a::GFP;rol-6]	This study
CL2166	N2	Is[Pgst-4::GFP]	(Link & Johnson 2002)
CF1533	N2	muIs84 [Psod-3::GFP]	(Libina et al. 2003)

The cgef-1(gk261) mutant was outcrossed at least 4 times with wild-type N2.

Supplementary Table 5: List of plasmids used for expression in cell culture.

Plasmid	Details
N-terminally Flag tagged pcDNA6. CeRHEB-1	Full length coding region of C. elegans RHEB-1 subcloned into pcDNA6 with N-terminal Flag-tag
N-terminally V5 tagged pcDNA6. CGEF-1a	Coding region of C. elegans cgef-1a subcloned into pcDNA6 with N-terminal V5-tag
N-terminally Flag tagged pcDNA6. humanRheb	Full length coding region of human Rheb subcloned into pcDNA6 with N-terminal Flag-tag
N-terminally Flag tagged pcDNA6. human CD2AP	Full length coding region of human CD2AP subcloned into pcDNA6 with N-terminal Flag-tag
N-terminally Flag tagged pcDNA6. Rab23l	Coding region of full length mouse Rab23 subcloned into pcDNA6 with N-terminal Flag-tag
N-terminally V5 tagged pcDNA6. proto-Dbl	Coding region of full length Dbl (residues 1-925) subcloned into pcDNA6 with N-terminal V5-tag
N-terminally V5 tagged pcDNA6. onco-Dbl	Coding region of Dbl (residues 498-925) subcloned into pcDNA6 with N-terminal V5-tag

Supplementary Table 6: List of primers used for RT-PCR

Target gene	Forward primer 5′ to 3′	Reverse primer 5′ to 3′
gst-4	ATGCTCGTGCTCTTGCTGAG	GACTGACCGAATTGTTCTCCAT
F20D6.11	GGAAATTCTCGGTAGAATCGAA	ACGACTACGAACTTCGAACA
gcs-1	AATCGATTCCTTTGGAGACC	ATGTTTGCCTCGACAATGTT
nit-1	AATCCTCCGACTATCCCTTG	AGCGAATCGTTTCTTTTGTG
sod-3	CTCCAAGCACACTCTCCCAG	ACCGAAGTCGCGCTTAATAGT
hsp-12.6	GGAGTTGTCAATGTCCTCGACG	GAAGTTCTCCAATGTTCTTGAC
mtl-1	GCAAGTGTGACTGCAAAA	AGTCTCCCTTACATCCAG
cdc-42	CTGCTGGACAGGAAGATTACG	CTCGGACATTCTCGAATGAAG
Y45F10D.4	GTCGCTTCAAATCAGTTCAGC	GTTCTTGTCAAGTGATCCGAC
