# Supplementary material for: CGEF-1 regulates mTORC1 signaling during adult longevity and stress response in C. elegans
Source: Oncotarget. 2018 Jan 6;9(11):9581–95. doi: 10.18632/oncotarget.24039 (PMC5839386; doi:10.18632/oncotarget.24039)
Supplement: Supplementary file 2 [file oncotarget-09-9581-s002.docx]

**Supplementary Table S1: Individual lifespan analyses**

| **Strain** | **RNAi** | **Mean lifespan ± SEM** | **Median** | **75%ile** | ***P* value vs N2** | ***P* value vs control** | **% change** | **N** |
| --- | --- | --- | --- | --- | --- | --- | --- | --- |
|  |  |  |  |  |  |  |  |  |
|  |  |  |  |  |  |  |  |  |
| N2 |  | 18.5 ± 0.3 | 19 | 21 |  |  |  | 80/85 |
| *cgef-1* |  | 21.3 ± 0.3 | 21 | 23 | <0.0001 |  | 15% | 71/75 |
| N2 |  | 17.2 ± 0.4 | 17 | 19 |  |  |  | 59/73 |
| *cgef-1* |  | 19.6 ± 0.5 | 19 | 22 | 0.002 |  | 13% | 58/73 |
| N2 |  | 16.8 ± 0.4 | 16 | 19 |  |  |  | 77/80 |
| *cgef-1* |  | 18.6 ± 0.5 | 18 | 21 | 0.017 |  | 11% | 55/79 |
| N2 | control | 18.4 ± 0.4 | 18 | 21 |  |  |  | 69/80 |
| N2 | *cgef-1* | 21.1 ± 0.5 | 21 | 24 | <0.0001 |  | 15% | 66/80 |
| *skn-1(zu67)* | control | 11.5 ± 0.4 | 12 | 14 | <0.0001 |  | -37% | 63/78 |
| *skn-1(zu67)* | *cgef-1* | 13.3 ± 0.5 | 13 | 15 | <0.0001 | <0.01^g^; | -28% | 65/80 |
|  |  |  |  |  |  | <0.0001^b^ |  |  |
| N2 | control | 18.3 ± 0.3 | 18 | 19 |  |  |  | 78/80 |
| N2 | *cgef-1* | 20.6 ± 0.4 | 20 | 23 | <0.0001 |  | 13% | 76/80 |
| *skn-1(zu67)* | control | 15.4 ± 0.3 | 16 | 17 | <0.0001 |  | -16% | 66/67 |
| *skn-1(zu67)* | *cgef-1* | 17.0 ± 0.5 | 18 | 19 |  | <0.001^g^; <0.0001^b^ | -7% | 41/50 |
| *daf-16(mgDf47)* | control | 15.4 ± 0.4 | 15 | 18 | <0.0001 |  | -16% | 81/81 |
| *daf-16(mgDf47)* | *cgef-1* | 15.2 ± 0.4 | 15 | 17 | <0.0001 | <0.0001^b^; | -17% | 81/85 |
|  |  |  |  |  |  | ns^h^ |  |  |
| N2 | control | 18.5 ± 0.3 | 19 | 21 |  |  |  | 80/82 |
| *cgef-1 (gk261)* | control | 21.3 ± 0.3 | 21 | 23 | <0.0001 |  | 15% | 72/75 |
| N2 | *daf-15* | 21.4 ± 0.4 | 21 | 24 | <0.0001 |  | 16% | 74/82 |
| N2 | *let-363* | 23.4 ± 0.5 | 23 | 25 | <0.0001 |  | 27% | 73/78 |
| *cgef-1(gk261)* | *daf-15* | 22.9 ± 0.4 | 23 | 26 | <0.0001 | <0.001^c^; | 24% | 72/80 |
|  |  |  |  |  |  | ns^e^ |  |  |
| *cgef-1(gk261)* | *let-363* | 22.6 ± 0.4 | 23 | 25 | <0.0001 | <0.01^c^; | 22% | 75/83 |
|  |  |  |  |  |  | ns^f^ |  |  |
| N2 | control | 17.8 ± 0.4 | 18 | 20 |  |  |  | 66/71 |
| *cgef-1(gk261)* | control | 19.7 ± 0.4 | 19 | 22 | <0.01 |  | 11% | 67/74 |
| N2 | *rheb-1* | 19.3 ± 0.3 | 19 | 21 | <0.01 |  | 8% | 65/73 |
| *cgef-1(gk261)* | *rheb-1* | 19.5 ± 0.4 | 19 | 22 | <0.01 | ns^c^; ns^d^ | 9% | 65/74 |
| N2 | *daf-15* | 21.4 ± 0.4 | 22 | 23 | <0.0001 |  | 20% | 70/78 |
| *cgef-1(gk261)* | *daf-15* | 21.6 ± 0.4 | 22 | 23 | <0.0001 | ns^e^ | 21% | 69/76 |
| N2 | *let-363* | 21.5 ± 0.4 | 22 | 24 | <0.0001 |  | 21% | 70/76 |
| *cgef-1(gk261)* | *let-363* | 21.9 ± 0.4 | 22 | 24 | <0.0001 | ns^f^ | 23% | 67/78 |
| N2 | control | 16.9 ± 0.4 | 17 | 19 |  |  |  | 70/71 |
| *cgef-1(gk261)* | control | 19.4 ± 0.4 | 19 | 22 | <0.0001 |  | 15% | 66/70 |
| N2 | *rheb-1* | 19.1 ± 0.4 | 19 | 21 | <0.001 |  | 13% | 68/79 |
| *cgef-1(gk261)* | *rheb-1* | 19.0 ± 0.5 | 19 | 21 | <0.0001 | ns^c^; ns^d^ | 12% | 70/83 |
| N2 | *daf-15* | 20.4 ± 0.5 | 20 | 23 | <0.0001 |  | 21% | 67/77 |
| *cgef-1(gk261)* | *daf-15* | 20.5 ± 0.6 | 20 | 24 | <0.0001 | ns^c^; ns^e^ | -21% | 64/74 |
| N2 | *let-363* | 20.7 ± 0.5 | 20 | 24 | <0.0001 |  | 23% | 67/84 |
| *cgef-1(gk261)* | *let-363* | 20.7 ± 0.5 | 21 | 24 | <0.0001 | ns^f^; ns^c^ | 23% | 66/72 |
| *Ex[unc-119]* | control | 24,3 ± 0.5 | 25 | 27 |  |  |  | 67/70 |
| *Ex[rheb-1::GFP,*  *unc-119(+)]* | control | 18.7 ± 0.8 | 17 | 24 | <0.0001 |  | -23% | 52/58 |
| *Ex[unc-119]* | *cgef-1* | 25.0 ± 0.6 | 26 | 29 | ns |  | 3% | 68/72 |
| *Ex[rheb-1::GFP;*  *unc-119(+)]* | *cgef-1* | 18.6 ± 0.7 | 18 | 20 | <0.0001 | ns^k^ | -24% | 53/62 |
| *Ex[unc-119]* | control | 22.5 ± 0.5 | 23 | 25 |  |  |  | 63/68 |
| *Ex[rheb-1::GFP,*  *unc-119(+)]* | control | 17.6 ± 0.6 | 17 | 21 | <0.0001 |  | -22% | 58/68 |
| *Ex[unc-119]* | *cgef-1* | 25.4 ± 0.5 | 26 | 28 | <0.0001 |  | 13% | 60/70 |
| *Ex[rheb-1::GFP,*  *unc-119(+)]* | *cgef-1* | 17.9 ± 0.6 | 18 | 19 | <0.0001 | ns^k^ | -20% | 52/64 |
| N2 | control | 21.3 ± 0.4 | 22 | 23 |  |  |  | 70/74 |
| N2 | *cgef-1* | 24.7 ± 0.5 | 25 | 27 | <0.0001 |  | 16% | 71/72 |
| *daf-16(mgDf47)* | control | 18.7 ± 0.5 | 19 | 21 | <0.0001 |  | -12% | 66/67 |
| *daf-16(mgDf47)* | *cgef-1* | 17.8 ± 0.5 | 18 | 21 | <0.0001 | ns^h^ | -16% | 60/63 |
| *skn-1(zu67)* | control | 15.6 ± 0.3 | 15 | 17 | <0.0001 |  | -26% | 65/66 |
| *skn-1(zu67)* | *cgef-1* | 17.8 ± 0.3 | 18 | 20 | <0.0001 | <0.0001^b^ | -16% | 64/69 |
| *daf-16;skn-1* | control | 14.8 ± 0.3 | 15 | 17 | <0.0001 |  | -30% | 60/61 |
| *daf-16;skn-1* | *cgef-1* | 15.4 ± 0.3 | 15 | 17 | <0.0001 | ns^i^ | -27% | 67/67 |
| N2 | control | 18.8 ± 0.4 | 19 | 21 |  |  |  | 69/71 |
| N2 | *cgef-1* | 22.1 ± 0.5 | 22 | 25 | <0.0001 |  | 17% | 62/65 |
| *skn-1(zu67)* | control | 15.2 ± 0.4 | 15 | 18 | <0.0001 |  | -19% | 59/59 |
| *skn-1(zu67)* | *cgef-1* | 17.2 ± 0.5 | 18 | 20 |  | <0.0001^b^ | -9% | 57/59 |
| *daf-16(mgDf47)* | *control* | 17.3 ± 0.5 | 17 | 20 | <0.01 |  | -8% | 65/66 |
| *daf-16(mgDf47)* | *cgef-1* | 17.7 ± 0.5 | 18 | 20 | ns | <0.0001^b^; ns^h^ | -6% | 67/68 |
| *daf-16(mgDf47);* | *control* | 13.5 ± 0.5 | 13 | 16 | <0.0001 |  | -28% | 59/59 |
| *skn-1(zu67)* |  |  |  |  |  |  |  |  |
| *daf16(mgDf47);* | *cgef-1* | 13.0 ± 0.4 | 13 | 16 | <0.0001 | ns^i^ | -30% | 71/71 |
| *skn-1(zu67)* |  |  |  |  |  |  |  |  |
| N2 | control | 20.5 ± 0.4 | 20 | 23 |  |  |  | 56/57 |
| N2 | cgef-1 | 23.7 ± 0.5 | 24 | 27 | <0.0001 |  | 15% | 53/55 |
| *skn-1(zu67)* | control | 15.7 ± 0.5 | 16 | 18 | <0.0001 |  | -23% | 36/37 |
| *skn-1(zu67)* | cgef-1 | 19.0 ± 0.4 | 19 | 21 | <0.01 | <0.0001^b^ | -7% | 42/42 |
| *daf-16(mgDf47)* | control | 18.0 ± 0.5 | 18 | 20 | <0.001 |  | -12% | 55/56 |
| *daf-16(mgDf47)* | cgef-1 | 18.1 ± 0.5 | 18 | 21 | <0.01 | ns^h^ | -12% | 48/52 |
| *daf-16;skn-1* | control | 14.6 ± 0.4 | 14 | 16 | <0.0001 |  | -28% | 43/43 |
| *daf-16;skn-1* | *cgef-1* | 15.0 ± 0.4 | 15 | 17 | <0.0001 | ns^i^ | -27% | 45/45 |

These experiments correspond to the composites shown in Table 1. Lifespan extensions correspond to parallel wild-type N2 control experiments. N represents total number of animals dying of old age versus those in total experiment. SEM = standard error of the mean. ns = not significant.

*p*-values (log-rank test) refer to the following control experiments:

^a^ N2 wild type,

^b^ *cgef-1(RNAi)*

^c^ *cgef-1(gk261);control*(RNAi),

^d^ *rheb-1*(RNAi),

^e^ *daf-15/Raptor*(RNAi),

^f^ *let-363/CeTOR*(RNAi),

^g^ *skn-1(zu67);control*(RNAi),

^h^ *daf-16(mgDf47);control*(RNAi),

^i^ *daf-16(mgDf47);skn-1(zu67);control*(RNAi),

^k^ *Ex[rheb-1::GFP;unc-119(+)];control*
